# Epidemiological trend comparison and incidence prediction for elderly lung cancer in 204 countries and territories: a retrospective study

**DOI:** 10.1016/j.clinsp.2026.101161

**Published:** 2026-10-15

**Authors:** Songjing Chen, Sizhu Wu

**Affiliations:** Institute of Medical Information, Chinese Academy of Medical Sciences & Peking Union Medical College, Beijing, China

**Keywords:** Lung cancer, Elderly, Incidence, Mortality, Prediction

## Abstract

**Background:**

Lung cancer is widely prevalent worldwide at present, especially in the elderly population aged over 65-years. It is essential to timely understand epidemiological trends of elderly lung cancer and project the future incidence in global and regions, which could be conducive to reducing the incidence and mortality of lung cancer.

**Methods:**

We stratified the large-scale population through ages, genders, regions and countries and territories. Lung cancer incidence, mortality and Disability Adjusted Life Years (DALYs), with 95% uncertainty intervals, were separately computed in global, regional and national levels. Average Annual Percentage Changes (AAPCs) were calculated through join point regression model to assess the temporal trend of each year. We employed Deep Neural Networks (DNN) for predicting the global and regional incidence of lung cancer to 2050 by gender, age and socio-demographic index.

**Results:**

Lung cancer incidence, mortality and DALYs for the elderly people were derived in the global and Socio-Demographic Index (SDI) levels, regional levels and national levels through the stratification of age and gender. We predicted lung cancer incidence of the elderly through DNN model by the year of 2050. Lung cancer incidence of the elderly men was decreased nearly 11% from 1990 to 2021 globally, with the AAPC value of -0.36% (95% CI -0.42% ‒ -0.30%). Elderly lung cancer incidence in high SDI region had significant drop of 21% from 1990 to 2021. The prediction indicated the decline trend in incidence of elderly lung cancer worldwide. Elderly lung cancer incidence in the high SDI region would have obviously decrease by 2050, whereas the high-middle SDI region would have highest lung cancer incidence in the elderly than other SDI regions by 2050.

**Conclusions:**

This research analysed the epidemiological trends of lung cancer for the elderly in global, regional and national levels and predicted the incidence trend of elderly lung cancer by the year of 2050. The findings could illuminate the trend of incidence, mortality and DALYs for elderly lung cancer and contribute to lung cancer early diagnosis and treatment in the elderly.

## Highlights

• Elderly men lung cancer incidence decreases 11% 1990‒2021 globally.
• Elderly incidence in high socio-demographic index region drops 21% 1990‒2021.
• Elderly lung cancer incidence displays the decline trend worldwide.

## Background

Lung cancer is widely prevalent worldwide at present, especially in the elderly population aged over 65-years. In the past decades, the incidence rate of lung cancer was high, accompanied by high mortality and disability rates, which affected the improvement of life quality of the elderly.^1^ It is essential to timely understand epidemiological trend of elderly lung cancer and project the future incidence in global and regions, which could be conducive to reducing the incidence and mortality of lung cancer, and diminishing the burden of prevention, control, diagnosis and treatment of lung cancer in countries and regions.

Several related researches were developed to analyze epidemiological trend of lung cancer. Epidemiological trends of lung cancer attributed to residential radon exposure were involved and found that rising absolute burdens of lung cancer underscored radon's persistent threat.^2^ Evolving trends of lung cancer were analyzed to explore the epidemiological trends, molecular diagnostics and therapeutic options primarily in India.^3^ Lung cancer incidence and mortality in the elderly people were more significant than other age groups.^4^^,^^5^ In order to explore lung cancer epidemiological trends for the elderly people in global and different regional levels, we conducted global, regional and national analysis of lung cancer incidence, mortality and Disability Adjusted Life Years (DALYs) for the elderly over 65-years. Yamaguchi T and colleague employed mathematical model to predict epidemiological trends of lung cancer by age and gender.^6^ Lung cancer incidence, mortality and prevalence were projected based on the “stock and flow” model to support health services planning in Denmark.^7^ Bayesian age-period-cohort analysis was proposed to predict lung cancer incidences in China and the United States to 2032.^8^ Random forest,^9^ Bayesian network.^10^ and other models.^11^, ^12^, ^13^ were leveraged to predict lung cancer incidence in specific countries or regions. We explored Deep Neural Networks (DNN) model to predict lung cancer incidence for global and Socio-Demographic Index (SDI) regions to the year of 2050. Moreover, both epidemiological trends analysis of elderly lung cancer of past thirty years and incidence projection by 2050 attempted to provide new insight for lung cancer prevention and control.

The aim of this research was to analyze and compare the epidemiological trends of lung cancer for the elderly in 204 countries and territories from 1990 to 2021 and predict lung cancer incidence for the elderly from 2022 to 2050. Through stratified the population into global, regional and national levels, we explored the epidemiological trends of lung cancer incidence, mortality and Disability Adjusted Life Years (DALYs) in elderly aged over 65-years and predicted the incidence of elderly lung cancer by the year of 2050. The findings of this research could help to provide evidence to prevent lung cancer occurrence and reduce lung cancer mortality and disability rate, which might illuminate precise clues for alleviating the global burden of elderly lung cancer.

## Methods

### Data sources

Lung cancer incidence, mortality and disability related data from the year of 1990 and 2021 were derived from Global Burden of Disease (GBD) study,^14^ which involved 204 countries and territories. Multiple dimensions were adopted in this research, such as gender, age, location, year, air pollution, ambient particulate matter pollution, household air pollution from solid fuels, smoking, dietary risk, residential radon, secondhand smoke and occupational exposure to asbestos, and so on. Samples with symptom of cough, chest pain, hemoptysis or dyspnea, which was characterized by abnormal cell growth in the trachea, bronchus, or lung tissues, and diagnostic code ICD10 (10th revision of International Classification of Disease): C34, were considered as lung cancer patients in this research. The retrospective study, which contented disease related data over thirty years and covered >200 countries and territories, didn’t included personally identifiable information. The study was conducted in accordance with the Declaration of Helsinki.

### Data preprocessing and stratifying

Because of the existence of missing values, abnormal values, white noises and other problem data, we conducted raw data preprocessing at first. We adopted multiple imputation method.^15^ to fill in the gap values, and the average value and the linear regression were respectively calculated for iterative imputation of the missing values. The noise smoothing technique such as singular value decomposition was applied to reduce white noises. The data of each follow-up year was represented in a matrix form at first. We conducted singular value decomposition for this matrix, and threshold processing of singular values was performed. We retained about 40 singular values. Energy proportion method was employed to validate. We calculate the proportion of the square sum of retained singular values to the square sum of all singular values. This proportion was 90%. We reconstructed the matrix through retained singular values and corresponding left and right singular vectors, and obtained the denoised data. Then we computed the age standardized incidence of lung cancer through Equation.^1^ The average annual percentage change (AAPC) of lung cancer incidence was calculated through Equation.^2^ Specifically, *k* was the total researched years, $w_{i}$ was the weight of the *i*th year in total years, $\beta_{i}$ was the regression coefficient of the *i*th year. Similarly, the age standardized mortality and the age standardized DALYs were computed, and the AAPC of lung cancer mortality and AAPC of DALYs were derived as well. We stratified the population through ages, genders, regions and countries and territories. The stratification analysis included gender (male, female and both), age (65‒69-years, 70‒74-years, 75‒79-years, 80‒84-years, 85‒89-years, 90‒94-years, older than 95-years and all ages) and locations (global, Sociodemographic Index (SDI) regions, geographical regions and countries).

(1) $$\text{Age}\;\text{stanstandardized}\;\text{incidence}=\frac{\sum \left(\text{Incidence}\;\text{rate}\;\text{of}\;\text{each}\;\text{age}\;\text{group}\times \text{Standardized}\;\text{population}\;\text{by}\;\text{age}\;\text{group}\right)}{\text{Total}\;\text{standardized}\;\text{population}}\times \text{Proportional}\;\text{base}$$

(2) $$\text{AAPC}=\left(\exp\left(\sum_{i=1}^{k}w_{i}\beta_{i}\right)-1\right)\times 100\%$$

### Lung cancer incidence prediction

We employed Deep Neural Networks (DNN) for predicting the global and regional incidence of lung cancer to 2050. DNN models of stratified population were trained and tested. The data from 1990 to 2018 were used for training DNN models, which were randomly divided in a ratio of 9:1, with 90% as the training set and 10% as the internal testing set. We adopted the 10-fold cross-validation to test the accuracy of DNN model. Then we applied the datasets from 2019 to 2021 for external testing to further validate the DNN models’ performance. One input layer, three hidden layers and one output layer were employed. The liner rectification function was used as activation function to improve DNN model’s representation capability. Mean squared error was the loss function and stochastic gradient descent function was used as the optimizer. The input features of DNN model included location, air pollution, ambient particulate matter pollution, household air pollution from solid fuels, smoking, dietary risk, residential radon, secondhand smoke and occupational exposure to asbestos and so on. Lung cancer incidence was predicted according to Eq. (3) through multiple iterations. *W_L_* was the weight matrix of input features in the *L*th layer of DNN model. *b_L_* was the bias vector corresponding to *W_L_. X* was the input features. Input nodes were 37, 20 nodes were involved in each hidden layer, and one output node was used. We set 0.01 as the initial learning rate and 18 as the batch size. Shapley Additive Explanations (SHAP) method was employed to improve the explainability of DNN model.

(3) $$y=f_{L-1}\left(W_{L-1}f_{L-2}\left(...f_{1}\left(W_{1}X+b_{1}\right)\right)+b_{L-1}\right)$$

### Statistical analysis

Lung cancer incidence, mortality and DALYs, with 95% uncertainty intervals, were separately computed in global, regional and national levels. AAPCs were calculated through join-point regression model to assess the temporal trend of each year,^16^ which represented the average change of lung cancer incidence, mortality or DALYs from the year of 1990 to 2021. Predicted lung cancer incidence through DNN model of stratified gender, age and SDI groups were analyzed. The statistical analysis in this research were conducted through R language version 4.3.3 and Python version 3.9.6. The research followed the CONSORT Statement.

## Results

### Global and SDI levels’ trends of the elderly lung cancer

#### Incidence

Through large scale global and SDI levels’ data analysis, the results indicated that the global epidemiologic trend of lung cancer incidence for both genders and all ages has shown decreasing from 1990 to 2021, especially in the male of all ages from 2011 to 2021. The AAPC of incidence in the male of all ages was −0.39% (95% CI −0.43% ‒ −0.31%) from 1990 to 2011, which had the value of −1.22% (95% CI −1.29% ‒ −1.16%) from 2011 to 2021, as shown in Fig. 1A. The peak lung cancer incidence of the male for all ages was deserved in 1990, which was 46 (95% CI 44‒49) per 100,000 population as listed in Table 1. However, in the elderly male (age older than 65-years), lung cancer incidence was 298 (95% CI 281‒315) per 100,000 in 1990, which was 266 (95% CI 232‒299) per 100,000 in 2021, with the AAPC value of −0.36% (95% CI −0.42% ‒ −0.30%) from 1990 to 2021 as shown in Table 1. Lung cancer incidence of the elderly female was 89 (95% CI 82‒95) per 100,000 in 1990 and 115 (95% CI 82‒95) per 100,000 in 2021, which has the AAPC value of 0.76% (95% CI 0.69%‒ 0.82%). The elderly lung cancer incidence in high SDI region was 271 (95% CI 257‒279) per 100,000 in 1990 and 215 (95% CI 192‒229), with the AAPC value of −0.41% (95% CI −0.55% ‒ −0.28%) from 1990 to 2021, which indicated the declined trend of elderly lung cancer incidence in high SDI region. AAPCs of elderly lung cancer incidence were 0.64% (95% CI 0.57%‒0.71%) in high-middle SDI region from 1990 to 2021, and 0.87% (95% CI 0.81%‒0.93%) in middle SDI region from 1990 to 2021, which reflected increased trend of elderly lung cancer incidence in high-middle SDI and middle SDI regions.

**Fig. 1:**
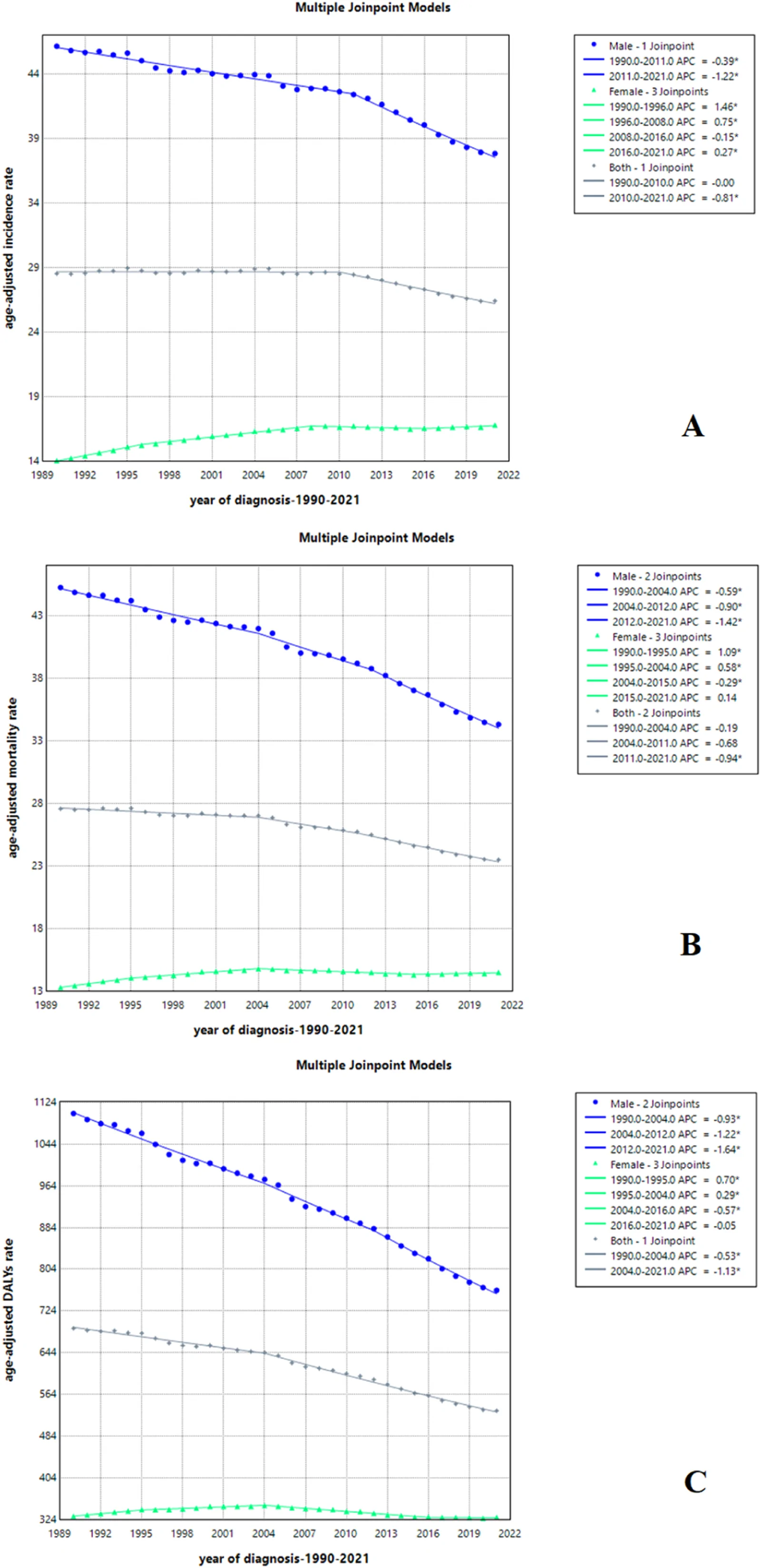
Average Annual Percentage Change (AAPC) and trends in male, female and both male and female (age-standardized) of lung cancer incidence, mortality and Disability Adjusted Life Years (DALYs) from the year of 1990 to 2021. (A) Lung cancer incidence; (B) Lung cancer mortality; (C) Lung cancer DALYs.

**Table 1:** Age standardized incidence and average annual percentage change (AAPC) of lung cancer at global and regional level from 1990 to 2021.

Group	Number of lung cancer in 1990 (thousands)	Age standardized rate of lung cancer in 1990 (per 100 000)	Number of lung cancer in 2021 (thousands)	Age standardized rate of lung cancer in 2021 (per 100 000)	AAPC 1990–2021 (%/year, 95% CI)
Global	1132 (1075‒1186)	28 (27.06‒29.91)	2281 (2063‒2510)	26 (23.90‒29.07)	−0.24 (−0.31- −0.17)
Global (age ≥ 65-years)	598 (563‒625)	181 (171‒191)	1505 (1344‒1651)	182 (163‒199)	0.47 (−0.02‒0.11)
Gender					
Male	833 (785‒886)	46 (44‒49)	1502 (1317‒1698)	38 (33‒43)	−0.59 (−0.66 ‒ −0.53)
Male (age ≥ 65-years)	426 (403‒450)	298 (281‒315)	982 (860‒1104)	266 (232‒299)	−0.36 (−0.42 ‒ −0.30)
Female	299 (277‒319)	14 (13‒15)	779(690‒862)	17(15‒19)	0.51 (0.41‒0.61)
Female (age ≥ 65-years)	171 (156‒182)	89 (82‒95)	524 (448‒584)	115 (99‒128)	0.76 (0.69‒0.82)
SDI level					
High	485 (467‒495)	44 (42‒45)	740 (676‒776)	34 (32‒36)	−0.72 (−0.86 ‒ −0.57)
High (age ≥ 65-years)	301 (285‒310)	271 (257‒279)	559 (501‒592)	215(192‒229)	−0.41 (−0.55 ‒ −0.28)
High-middle	366 (343‒388)	35 (34‒38)	731 (638‒838)	36 (32‒42)	0.06 (0.01‒0.11)
High-middle (age ≥ 65-years)	168 (157‒179)	203 (186‒217)	470 (408‒535)	264 (233‒298)	0.64 (0.57‒0.71)
Middle	217 (196‒240)	21 (19‒23)	649 (543‒753)	24 (20‒28)	0.50 (0.45‒0.54)
Middle (age ≥ 65-years)	100 (90‒110)	135 (122‒148)	398 (329‒462)	162 (134‒189)	0.87 (0.81‒0.93)
Low-middle	50 (44‒58)	8 (7‒9)	131 (119‒144)	9 (8‒10)	0.42 (0.40‒0.43)
Low-middle (age ≥ 65-years)	22 (19‒25)	53 (47‒61)	64 (58‒71)	57 (52‒63)	0.39 (0.37‒0.42)
Low	13 (11‒16)	6 (5‒7)	28 (24‒33)	6 (4‒7)	−0.14 (−0.25 ‒ −0.02)
Low (age ≥ 65-years)	6 (5‒7)	38 (32‒46)	13 (11‒15)	37 (31‒43)	−0.15 (−0.25 ‒−0.05)
Age					
65‒69	199 (189‒209)	168 (160‒176)	389 (351‒429)	139 (125‒153)	−0.50 (−0.54 ‒ −0.45)
70‒74	163 (155‒171)	199 (189‒208)	400 (360‒442)	192 (172‒212)	−0.13 (−0.23 ‒ −0.03)
75‒79	129 (122‒135)	216 (205‒223)	308 (274‒338)	231 (205‒254)	0.33 (0.19‒0.47)
80‒84	70 (64‒73)	202 (186‒214)	218 (187‒241)	246 (211‒273)	0.86 (0.68‒1.03)
85‒89	28 (24‒30)	193 (169‒202)	131 (109‒145)	283 (236‒313)	1.49 (1.32‒1.66)
90‒94	7 (6‒8)	167 (139‒182	49 (38‒54)	269 (210‒301)	1.83 (1.75‒1.92)
≥ 95	1 (1‒2)	112 (88‒127)	11 (8‒12)	191 (138‒221)	1.86 (1.78‒1.94)

#### Mortality

Consistent with lung cancer mortality trends as shown in Fig. 1B, mortality of the elderly lung cancer exhibited a decreasing trend in the whole elderly group and the elderly male group, and a slightly increasing trend in the elderly female group from 1990 to 2021, as displayed in Supplementary Table 1. Mortality of the elderly lung cancer in 1990 was 183 (95% CI 172‒191) per 100,000 people, and in 2021 was 174 (95% CI 153‒189) per 100,000 people, with the AAPC value of −0.26% (95% CI −0.33% ‒ −0.20%) from 1990 to 2021. Lung cancer mortality of the elderly male had significant decline trend over the past thirty years, which had AAPC value of −0.67% (95% CI −0.73% ‒ −0.60%) from 1990 to 2021. The mortality AAPC was −0.81% (95% CI −0.90% ‒ −0.71%) of the elderly lung cancer in high SDI region, whereas, in high-middle, middle and low-middle SDI regions, the AAPCs were 0.21% (95% CI 0.12%‒0.29%), 0.46% (95% CI 0.38%‒0.54%) and 0.37% (95% CI 0.35%‒0.40%) respectively. Therefore, lung cancer mortality of the elderly in high SDI regions had significantly decreased compared to high-middle, middle and low-middle regions.

#### DALYs

DALYs rate of the whole elderly group was 3695 (95% CI 3502‒3874) per 100,000 people in 1990, and this rate was 3065 (95% CI 2748‒3366) per 100,000 people in 2021, with the AAPC value of −0.50% (95% CI −0.56% ‒ −0.44%) from 1990 to 2021 as shown in Supplementary Table 1. The AAPC of DALYs rate of the elderly male was −0.87% (95% CI −0.95% ‒ −0.82%) over the past thirty years. And this A APC of the elderly female was 0.21% (95% CI 0.15% ‒ 0.27%) from 1990 to 2021, which represented that the DALYs of the elderly male had more obviously decline than the elderly female. Moreover, the AAPCs of DALYs rate of 65‒69-years elderly and 70‒74-years elderly were −0.89% (95% CI −0.94% ‒ −0.83%) and −0.52% (95% CI −0.63% ‒ −0.41%) respectively, as shown in Supplementary Table 1, which exhibited the significance decrease of lung cancer DALYs in 65‒74-years elderly from 1990 to 2021. The AAPC of DALYs rate of the elderly lung cancer in high SDI region was −1.12% (95% CI −1.21% ‒ −1.03%), which declined more obviously of lung cancer DALYs than high-middle, middle and low-middle regions as listed in Supplementary Table 1.

### Regional level trends of the elderly lung cancer

#### Incidence

Compared to the year of 1990, lung cancer incidence rates of the elderly displayed notable decreases in Central Latin America, High-income North America and Central Asia in 2021 as shown in Table 2, which had AAPC values of −1.61% (95% CI −1.71% ‒ −1.51%), −1.53% (95% CI −1.86% ‒ −1.19%) and −1.38% (95% CI −1.50% ‒ −1.27%) respectively. East Asia and Western Sub-Saharan Africa had experience significant lung cancer incidence increasing in the elderly people, with AAPC values of 1.04% (95% CI 0.83%‒1.25%) and 0.97% (95% CI 0.89%‒1.05%) from 1990 to 2021. Moreover, lung cancer incidence rate of the elderly male in East Asia was 394 (95% CI 287‒507) per 100,000 in 2021 as listed in Supplementary Table 2, which was higher 133% than the elderly female in East Asia 169 (95% CI 129‒213) per 100,000. In Central Latin America, High-income North America and Central Asia, lung cancer incidences of the elderly male were also higher than the elderly female in 2021.

**Table 2:** Age standardized incidence and AAPC of lung cancer in elderly (age over 65-years) at regional levels from 1990 to 2021.

Region	Cases in 1990 (thousands)	Age standardized rate in 1990 (per 100,000)	Cases in 2021 (thousands)	Age standardized rate in 2021 (per 100,000)	AAPC 1990–2021 (%/year, 95% CI)
Andean Latin America	1.40 (1.21‒1.60)	89 (78‒96)	3.77 (2.95‒4.63)	71 (55‒87)	−0.69 (−0.89 ‒ −0.49)
Australasia	5.79 (5.38‒6.14)	266 (248‒282)	12.3 (10.6‒13.7)	191 (162‒215)	−0.80 (−0.92 ‒ −0.68)
Caribbean	3.40 (3.19‒3.62)	147 (138‒157)	7.11 (6.26‒8.05)	139 (121‒158)	0.16 (0.04‒0.29)
Central Asia	4.33 (4.09‒4.55)	137 (130‒144)	4.85 (4.36‒5.32)	82 (74‒90)	−1.38 (−1.50 ‒ −1.27)
Central Europe	25.4 (24.4‒26.4)	187 (179‒195)	53.3 (49.3‒60.0)	206 (188‒222)	0.23 (0.12‒0.34)
Central Latin America	6.11 (5.83‒6.31)	98 (94‒101)	14.4 (12.7‒16.2)	64 (57‒72)	−1.61 (−1.71‒ −1.51)
Central Sub-Saharan Africa	0.91 (0.63‒1.39)	65.1 (47.1‒96.6)	1.93 (1.40‒2.88)	60.2 (45.1‒87.5)	−0.19 (−0.45‒ −0.07)
East Asia	135 (117‒154)	207 (180‒234)	630 (512‒753)	272 (217‒330)	1.04 (0.83‒1.25)
Eastern Europe	40.3 (38.9‒41.6)	169 (163‒175)	47.0 (42.9‒50.8)	126 (113‒138)	−1.15 (−1.25 ‒ −1.04)
Eastern Sub-Saharan Africa	2.29 (1.94‒2.74)	46.4 (39.7‒54.6)	4.60 (3.97‒5.36)	42.0 (36.6‒48.4)	−0.43 (−0.52‒ −0.33)
High-income Asia Pacific	40.3 (37.3‒42.3)	209 (195‒221)	134 (114‒146)	189 (155‒210)	−0.31 (−0.45‒ −0.18)
High-income North America	131 (122‒135)	360(339‒373)	188 (168‒199)	237 (211‒252)	−1.53 (−1.86‒ −1.19)
North Africa and Middle East	12.8 (10.5‒15.5)	111 (92.7‒133)	37.0 (31.9‒43.2)	109 (95‒128)	0.05 (−0.07‒ 0.16)
Oceania	213 (156‒310)	117 (88‒166)	570 (424‒816)	122 (93‒172)	0.18 (0.13‒0.23)
South Asia	13.7 (11.7‒16.0)	36.6 (31.6‒42.3)	45.9 (38.6‒52.3)	39.2 (33.2‒44.6)	0.03 (−0.10‒ 0.15)
Southeast Asia	21.8 (18.9‒24.7)	124 (109‒140)	67.7 (55.4‒78.1)	131 (107‒151)	−0.07 (−0.18‒0.05)
Southern Latin America	6.50 (6.04‒6.99)	158 (147‒170)	11.0 (9.93‒12.2)	126 (113‒139)	−0.53 (−0.64‒ −0.42)
Southern Sub-Saharan Africa	1.86 (1.60‒2.21)	93.7 (81.7‒110)	4.83 (4.36‒5.38)	111 (101‒123)	0.51 (0.20‒0.83)
Tropical Latin America	6.82 (6.42‒7.14)	100 (94.7‒105)	22.2 (20.0‒23.7)	93.6 (85.1‒100)	−0.22 (−0.32‒ −0.11)
Western Europe	136 (129‒140)	221 (210‒228)	211 (190‒225)	187 (166‒200)	−0.41 (−0.50‒ −0.32)
Western Sub-Saharan Africa	1.49 (1.24‒1.76)	23.6 (19.9‒27.7)	3.80 (3.24‒4.47)	29.5 (25.4‒34.4)	0.97 (0.89‒1.05)

#### Mortality and DALYs

Lung cancer mortality of the elderly in the High-income North America, Australasia and Eastern Europe showed a significant decreasing trend from 1990 to 2021, with AAPC values of −1.76% (95% CI −2.05% ‒ −1.47%), −1.45% (95% CI −1.53% ‒ −1.36%) and −1.42% (95% CI −1.54% ‒ −1.31%) respectively as listed in Supplementary Table 3. In Western Sub-Saharan Africa and East Asia, the mortality of elderly lung cancer had notable increase in 2021 compared to 1990, which had AAPC 0.97% (95% CI 0.89%‒1.05%) and 0.44% (95% CI 0.21%‒0.68%). Whereas in High-income North America in the year 2021, DALYs of elderly lung cancer decreased from 6232 (95% CI 5927‒6432) per 100,000 people to 3425 (95% CI 3102‒3617) per 100,000 people, with the overall AAPC of −2.15% (95% CI −2.42% ‒ −1,88%). And DALYs of elderly lung cancer in Central Latin America and Australasia also decreased from 1990 to 2021, with AAPC values of −1.84% (95% CI −1.94% ‒ −1.73%) and −1.82% (95% CI −1.89% ‒ −1.75%).

### National trends of the elderly lung cancer

#### Incidence

As shown in Supplementary Table 4, lung cancer incidence in aged over 65-years of several high incidence rate countries in 1990 significantly decreased in 2021 through comparing to the year of 1990. The elderly lung cancer incidence rate in 1990 of Canada, Netherlands, United States of America and Turkey were separately 236 (95% CI 205‒266) per 100,000, 220 (95% CI 191‒247) per 100,000, 236 (95% CI 211‒252) per 100,000 and 201 (95% CI 153‒256) per 100,000, and AAPC values of the elderly in these countries between 1990 and 2021 were separately −0.88% (95% CI −1.17% ‒ −0.59%), −0.83% (95% CI −0.95% ‒ −0.70%), −1.60% (95% CI −1.94% ‒ −1.25%) and −1.20% (95% CI −1.44% ‒ −0.97%), which reflected obvious decline of lung cancer incidence of the elderly in these countries. Lung cancer incidence of the elderly of 204 countries and territories in 2021 was shown in Fig. 2 and AAPC values of the elderly lung cancer incidence between 1990 and 2021 was shown in Fig. 3.

**Fig. 2:**
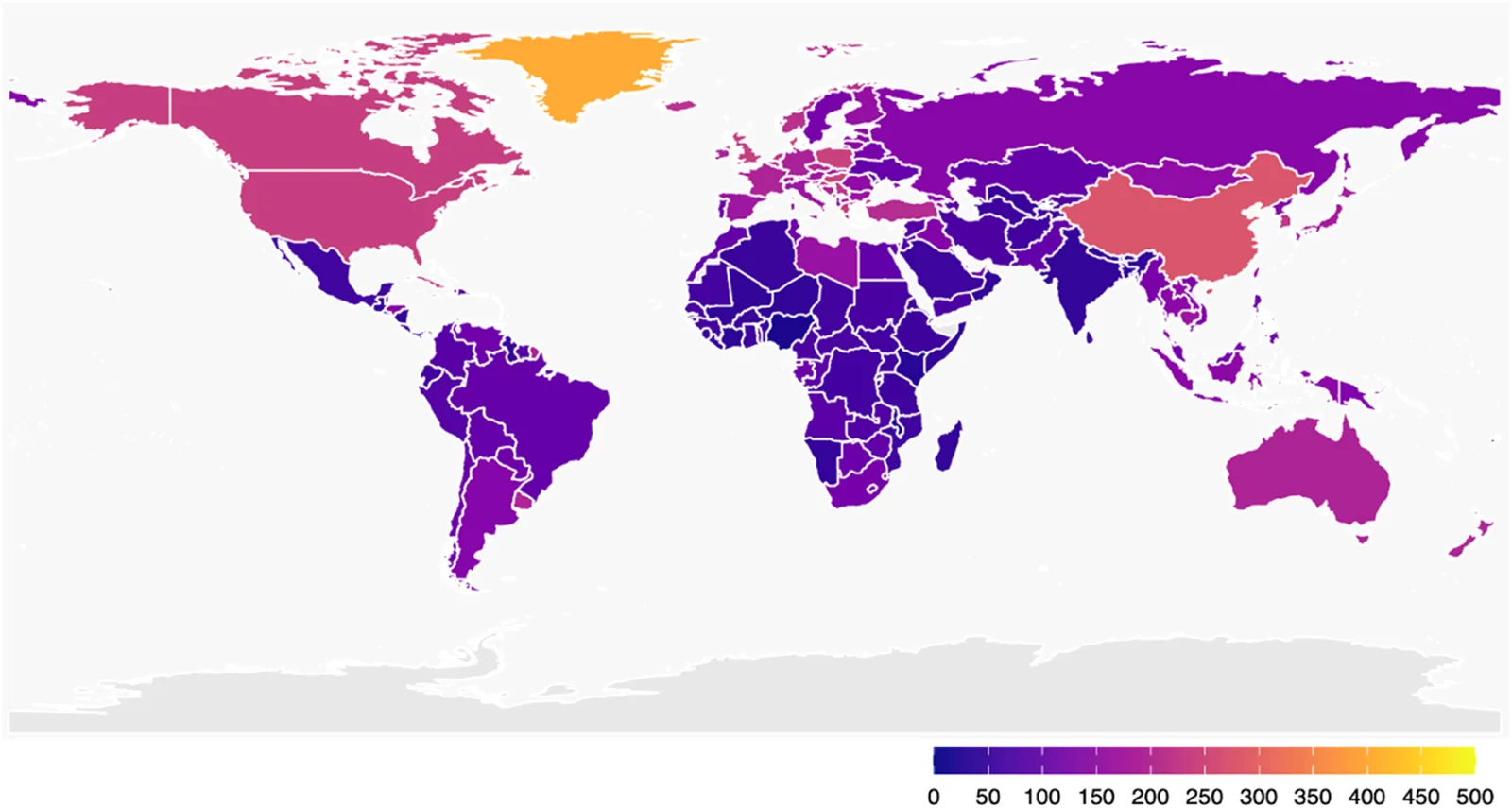
Lung cancer incidence of the elderly of 204 countries and territories in 2021 (per 100,000).

**Fig. 3:**
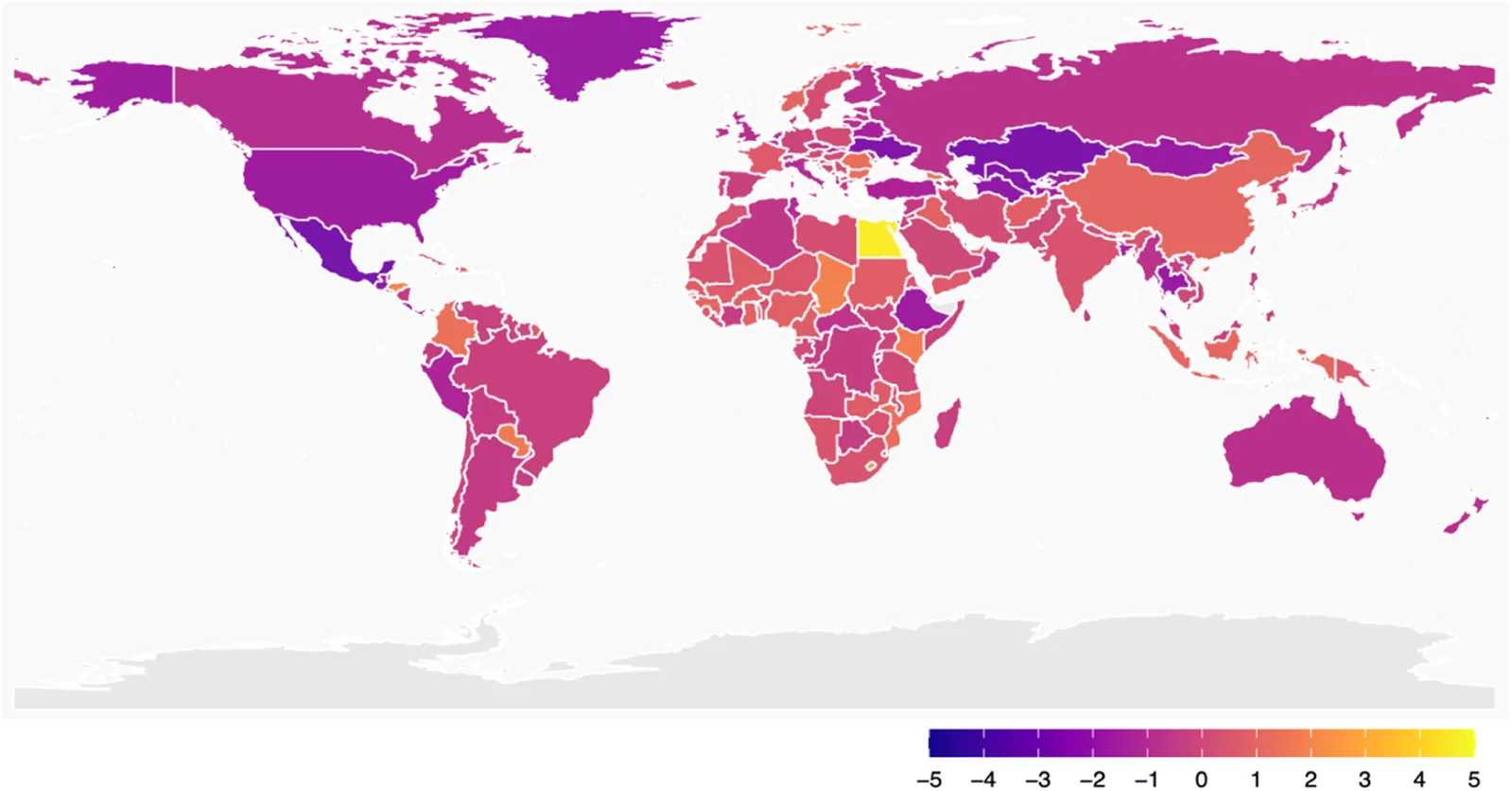
AAPC values of the elderly lung cancer incidence between 1990 and 2021 (%/year).

#### Mortality and DALYs

As shown in Supplementary Table 4, lung cancer mortality of the elderly in Australia, Finland, Singapore and United States of America had decreased AAPC values of the elderly, which were −1.43% (95% CI −1.53% ‒ −1.34), −1.30% (95% CI −1.43% ‒ −1.17%), −2.60% (95% CI −2.76% ‒ −2.43%) and −1.81% (95% CI −2.11% ‒ −1.50%). DALYs of elderly lung cancer were obviously declined in Singapore, United States of America, Australia and Finland from 1990 to 2021, with AAPC values of −3.02% (95% CI −3.19% ‒ −2.84%), −2.19% (95% CI −2.47% ‒ −1.92%), −1.82% (95% CI −1.89% ‒ −1.74%) and −1.51% (95% CI −1.68% ‒ −1.34%).

### Prediction of elderly lung cancer incidence

Elderly lung cancer incidences of the global and the SDI regions were predicted through DNN model by the year of 2050, as shown in Fig. 4. The result showed that lung cancer incidence of the aged over 65-years’ elderly would decrease from 2022 to 2050 in global level. The high-middle SDI region would have highest lung cancer incidence in the elderly by 2050. Moreover, the elderly men in the high-middle SDI region would have higher lung cancer incidence than the elderly women in the high-middle SDI region. Elderly lung cancer incidence in the high SDI region would have obviously decline by 2050. SHAP value of input features was derived as shown in Fig. 5, which demonstrated that smoking, air pollution and residential radon played important role in lung cancer occurrence.

**Fig. 4:**
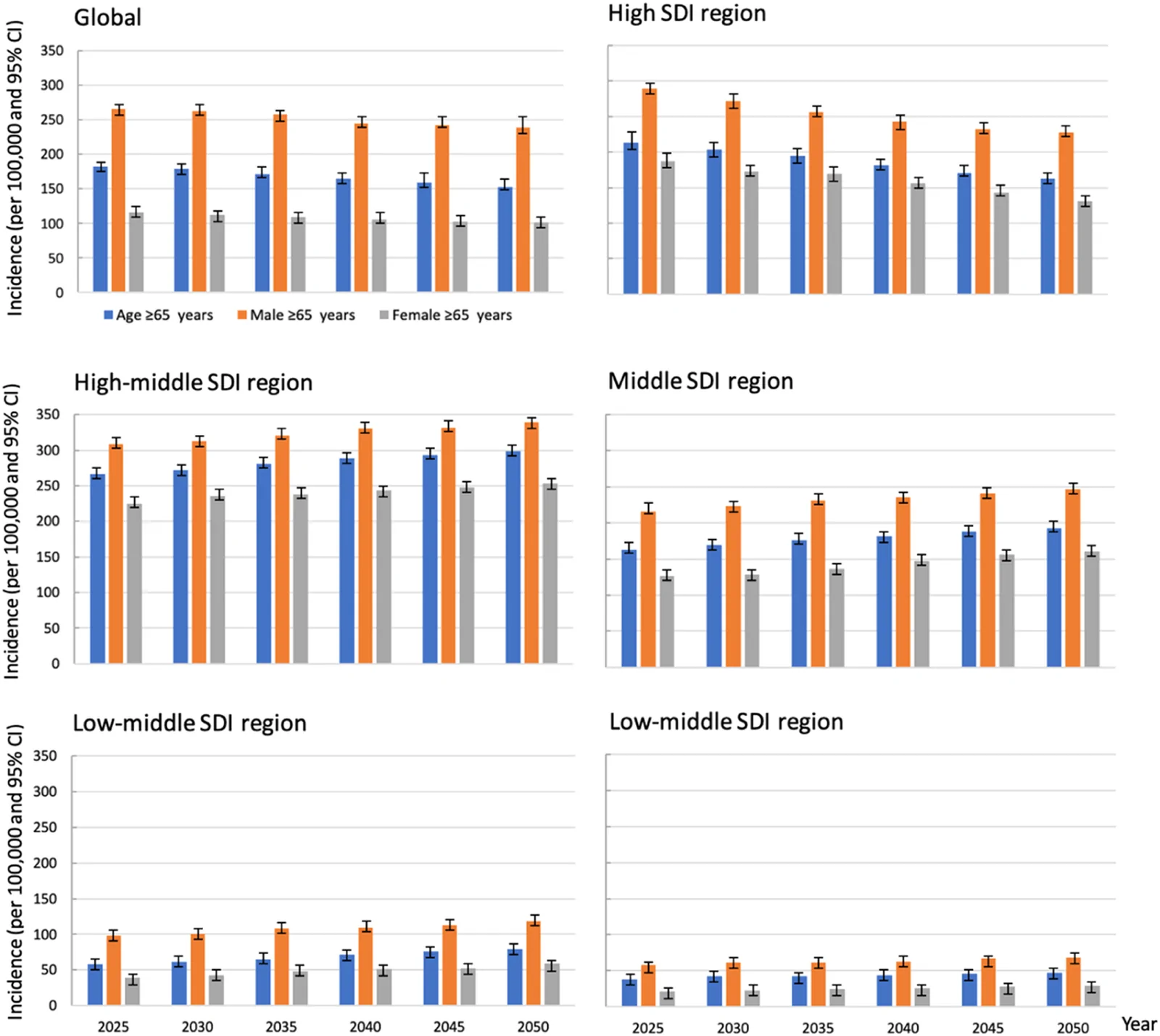
Prediction of elderly lung cancer incidence in global and SDI regions by 2050.

**Fig. 5:**
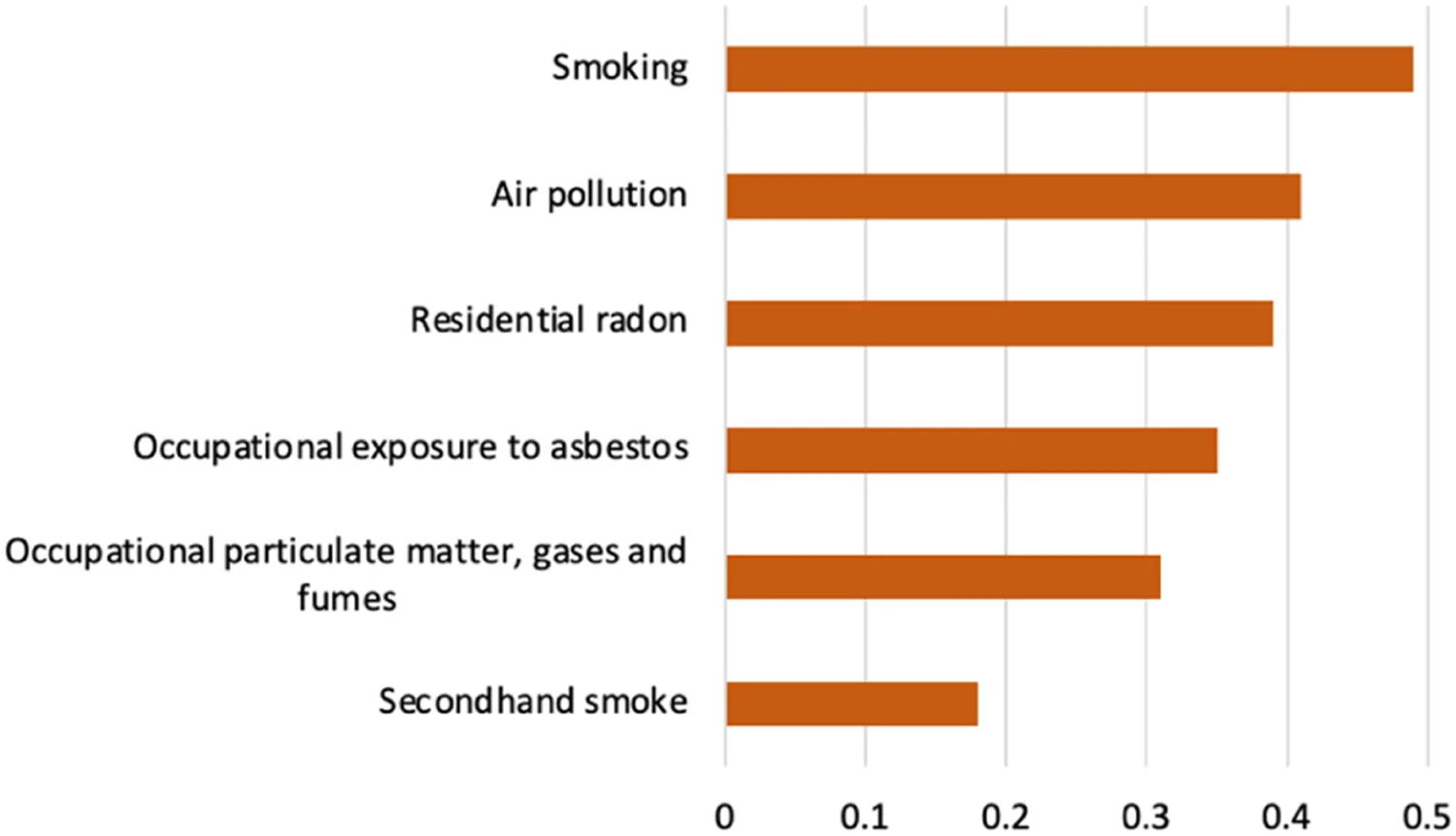
SHAP value of DNN model.

#### Model validation

DNN prediction model was trained through the data from 1990 to 2018. The 10-fold cross-validation was adopted to conduct internal validation. And the data from 2019 to 2021 were used for model’s external validation. Model performance of external validation and internal validation were shown in Table 3. Through external validation and internal validation, the R-squared (R^2^) of DNN models of the stratified elderly subgroups were close to 1, which were higher than 0.9.

**Table 3:** The performance of DNN models’ external and internal validation.

Subgroups	External validation			Internal validation		
	MSE^a^	RMSE	R^2^	MSE^a^	RMSE	R^2^
Age ≥65-years	1.236	1.085	0.927	1.287	1.061	0.932
Male ≥65-years	1.377	1.204	0.939	1.358	1.189	0.941
Female ≥65-years	1.532	1.176	0.931	1.543	1.123	0.929

MSE, Mean Squared Error; RMSE, Root Mean Squared Error; R^2^, R-squared.

### Comparison with other methods

Multiple regression and Bayesian network were adopted to compared with DNN-based method to predict lung cancer incidence of the elderly, which were mainly used in related projection studies of lung cancer incidence. We developed multiple regression models and Bayesian network models to predict elderly lung cancer incidence through GBD training dataset and internal validation dataset from 1990 to 2018. And the GBD data from 2019 to 2021 were leveraged for external validation. Lung cancer incidence trends of the elderly from 2022 to 2050, which were derived through multiple regression models and Bayesian network models, were mainly consistent with the DNN models. But MSEs of DNN models of external validation for stratified groups were all lower than multiple regression models and Bayesian network models. And the values of R^2^ of DNN models were closer to 1 than multiple regression models and Bayesian network models. Therefore, DNN models performed better than multiple regression models and Bayesian network models for lung cancer incidence prediction of the elderly (Table 4).

**Table 4:** Comparison of performance among multiple regression models, Bayesian network models and DNN models of external validation.

Subgroups	Multiple regression			Bayesian network			DNN		
	MSE	RMSE	R^2^	MSE	RMSE	R^2^	MSE	RMSE	R^2^
Age ≥65-years	3.569	3.315	0.752	2.651	2.496	0.812	1.236	1.085	0.927
Male ≥65-years	3.797	3.626	0.738	3.257	2.931	0.783	1.377	1.204	0.939
Female ≥65-years	3.983	3.622	0.743	3.672	3.064	0.792	1.532	1.176	0.931

MSE, Mean Squared Error; RMSE, Root Mean Squared Error; R^2^, R-squared.

## Discussion

We proposed and analyzed lung cancer incidence, mortality and DALYs for the elderly people in the global and SDI levels, regional levels and national levels through the stratification of age and gender. We predicted lung cancer incidence of the elderly through DNN model by the year of 2050. Lung cancer incidence of the elderly men was decreased nearly 11% from 1990 to 2021 globally, with the AAPC value of −0.36% (95% CI −0.42% ‒ −0.30%). Elderly lung cancer incidence in high SDI region had significant drop of 21% from 1990 to 2021. East Asia and Western Sub-Saharan Africa experienced lung cancer incidence increase in the elderly people from 1990 to 2021. Lung cancer mortality of the elderly in the High-income North America, Australasia and Eastern Europe showed a significant decreasing trend from 1990 to 2021, which might be due to the decrease in smoking prevalence.^17^, ^18^, ^19^ and air pollution.^20^^,^^21^ The government of United States took a series of policies for tobacco control from 1960s, which brought the declines of smokers’ number and lung cancer mortality in the country. The annual rate of decline in lung cancer mortality from 2% between 2005 and 2013 to 4% between 2013 and 2021 in the United States.^22^ The revised Ambient Air Quality Directive of European Commission in 2024 proposed that the annual average concentration limit of particulate matter 2.5 was decreased from 25 μg/m^3^ to 10 μg/m^3^, aligning with the WHO recommended value to significantly reduce the burden of respiratory diseases.^21^ Prediction results indicated the decline trend in lung cancer incidence of elderly worldwide. Elderly lung cancer incidence in the high SDI region would decrease obviously by 2050, whereas the high-middle SDI region would have highest lung cancer incidence in the elderly than other SDI regions by 2050. Furthermore, the elderly men in the high-middle SDI region would have higher lung cancer incidence than the elderly women in this region.

Lung cancer was predominantly the chronic disease of the elderly with about 50% of diagnoses in patients aged over 70-years and about 14% in those older than 80.^4^^,^^23^ Our main research subjects were elderly people, and we developed epidemiological trends comparison of lung cancer incidence, mortality and DALYs for the elderly in global, regional and national levels. Chouaid C and colleagues described epidemiology of metastatic lung cancer in France between 2013 and 2021 based on the French claims database.^24^ Mangone L explored changes in the histology of lung cancer in northern Italy and analyzed the impact on incidence and mortality.^25^ Specific country or region was selected to demonstrate epidemiology of lung cancer in the majority of related studies. Burkart KG and colleagues developed a comprehensive analysis of spatial patterns and temporal trends in exposure and attributable disease based on Bayesian algorithm.^26^ Random forest model integrating clinical, pathological, and genomic data was leveraged to predict secondary cancers’ incidence.^27^ Logistic regression model was constructed to compare treatment modalities offered to the aged over 70-years lung cancer patients with patients under 70 in Brazil.^28^ However, random forest,^29^ Bayesian network.^30^^,^^31^ and other algorithms.^32^, ^33^, ^34^ were adopted to analyze and project lung cancer incidence in recent researches. Through compared with Bayesian network and multiple regression, the proposed DNN model for predicting elderly lung cancer incidence to 2050 had high performance in stratified groups.

Several limitations were existed in this research. Firstly, retrospective data were used to explore epidemiological trends of lung cancer for the elderly. Data statistics exhibited a time lag, which would be continuously followed up in future work. Secondly, we mainly focused on prediction of the incidence of elderly lung cancer. However, the mortality and DALYs for elderly lung cancer could be projected to comprehensively analyze the future epidemiological trends of lung cancer for the elderly. Thirdly, epidemiological trends of elderly lung cancer were conducted based on large-scale population cohort, which lacked individual-specific projection of lung cancer incidence. We could explore the application of this prediction method to individuals level research of elderly lung cancer.

## Conclusions

This research analyzed and compared the epidemiological trends of lung cancer for the elderly in global, regional and national levels, and predicted the incidence of elderly lung cancer by the year of 2050. The findings could illuminate the incidence, mortality and DALYs for elderly lung cancer and contribute to lung cancer early diagnosis and treatment for the elderly.

## Ethics approval and consent to participate

Not applicable.

## Consent for publication

Not applicable.

## Funding

This study was supported by National Science and Technology Major Project for the Prevention and Treatment of Cancer, Cardiovascular, Respiratory, and Metabolic Diseases (Grant Number 2023ZD0509702), National Key Research and Development Program of China (Grant Number 2022YFF0712000), 10.13039/501100001809National Natural Science Foundation of China (NSFC) (Grant Number 62106286) and CAMS Innovation Fund for Medical Sciences (CIFMS) (Grant Number 2021-I2M-1–057).

## Conflicts of interest

The authors declare no conflicts of interest.

## Data Availability

The datasets used and analyzed during the current study are available from the corresponding author on reasonable request.

## References

[1] Ganti A.K., Klein A.B., Cotarla I., Seal B., Chou E. (2021). Update of incidence, prevalence, survival, and initial treatment in patients with non-small cell lung cancer in the US. JAMA Oncol.

[2] Xiong Q., Zhang Z., Peng J., Liang Y., Lian W., Zhao Y. (2025). Epidemiological trends of lung cancer attributed to residential radon exposure at global, regional, and national level: a trend analysis study from 1990 to 2021. Front Public Health.

[3] Deshpand R., Chandra M., Rauthan A. (2022). Evolving trends in lung cancer: epidemiology, diagnosis, and management. Indian J Cancer.

[4] Casaluce F., Sgambato A., Maione P., Spagnuolo A., Gridelli C. (2018). Lung cancer, elderly and immune checkpoint inhibitors. J Thorac Dis.

[5] Ju W., Zheng R., Zhang S., Zeng H., Sun K., Wang S. (2023). Cancer statistics in Chinese older people, 2022: current burden, time trends, and comparisons with the US, Japan, and the Republic of Korea. Sci China Life Sci.

[6] Yamaguchi T., Nishiura H. (2019). Predicting the epidemiological dynamics of lung cancer in Japan. J Clin Med.

[7] Jakobsen E., Olsen K.E., Bliddal M., Hornbak M., Persson G.F., Green A. (2021). Forecasting lung cancer incidence, mortality, and prevalence to year 2030. BMC Cancer.

[8] Xu M., Li M., Pei J., Wu J., Jiang Y., Jiang C. (2022). Gender disparities in incidence and projections of lung cancer in China and the United States from 1978 to 2032: an age-period-cohort analysis. Cancer Causes Control.

[9] Guo L.R., Tan J.F., Hughes M.C. (2025). Comparison of dynamic mode decomposition with other data-driven models for lung cancer incidence rate prediction. Front Public Health.

[10] Jiang Y., Han R., Su J., Fan X., Yu H., Tao R. (2022). Trends and predictions of lung cancer incidence in Jiangsu Province, China, 2009-2030: a Bayesian age-period-cohort modelling study. BMC Cancer.

[11] Kafle R.C., Kim D.Y., Holt M.M. (2023). Gender-specific trends in cigarette smoking and lung cancer incidence: a two-stage age-stratified bayesian joinpoint model. Cancer Epidemiol.

[12] Piva F., Tartari F., Giulietti M., Aiello E., Cheng L., Lopez-Beltran A. (2021). Predicting future cancer burden in the United States by artificial neural networks. Future Oncol.

[13] Machida H., Inoue S., Shibata Y., Kimura T., Ota T., Ishibashi Y. (2021). The incidence and risk analysis of lung cancer development in patients with chronic obstructive pulmonary disease: possible effectiveness of annual CT-screening. Int J Chron Obs Pulmon Dis.

[14] Institute for Health Metrics and Evaluation (2025). https://vizhub.healthdata.org/gbd-results/.

[15] Schafer J.L. (1999). Multiple imputation: a primer. Stat Methods Med Res.

[16] Kim H.J., Fay M.P., Feuer E.J., Midthune D.N. (2000). Permutation tests for joinpoint regression with applications to cancer rates. Stat Med.

[17] Guerra-Tort C., López-Vizcaíno E., Santiago-Pérez M.I., Rey-Brandariz J., Candal-Pedreira C., Ruano-Ravina A. (2024). Time dependence between tobacco consumption and lung cancer mortality in Spain. Arch Bronconeumol.

[18] Borg M., Tønnesen H., Ibsen R., Hilberg O., Løkke A. (2024). Lung cancer: a nationwide analysis of sex and age incidence trends from 1980 to 2022. Acta Oncol.

[19] Yang X.-R., Man J.-Y., Chen H., Zhang Q., Yin P., He J. (2021). Temporal trends of the lung cancer mortality attributable to smoking from 1990 to 2017: a global, regional and national analysis. Lung Cancer.

[20] Myers D., Mainali S., Afrin S., Kriebel D. (2026). Air pollution and lung cancer: an investigation of county-level residual incidence rates with smoking eliminated. Env Health.

[21] European Commission (2025). https://environment.ec.europa.eu/topics/air/air-quality_en.

[22] American Association for Cancer Research (AACR) (2024). Cancer in 2024 [M]. AACR Cancer Prog Rep.

[23] Xu J., Liao J.Y., Yan Q., Jiao Y., Hu Y., Zhang X. (2024). Trends analysis of cancer incidence, mortality, and survival for the elderly in the United States, 1975-2020. Cancer Med.

[24] Chouaid C., Marchal C., Apert M., Bensimon L., Guimard N., Née M. (2025). Epidemiology of metastatic lung cancer in France between 2013 and 2021: observational study using the French claims database. Bull Cancer.

[25] Mangone L., Marinelli F., Bisceglia I., Zambelli A., Zanelli F., Pagano L. (2023). Changes in the histology of lung cancer in northern Italy: impact on incidence and mortality. Cancers.

[26] GBD 2021 Household Air Pollution Collaborators (2025). Global, regional, and national burden of household air pollution, 1990-2021: a systematic analysis for the global burden of disease study 2021. Lancet.

[27] Sadeghi H., Seif F., Khanmohammadi S. (2025). Machine learning for early prediction of secondary cancer after radiotherapy. Sci Rep.

[28] Costa G.J., de Mello M.J.G., Ferreira C.G., Thuler L.C.S. (2017). Undertreatment trend in elderly lung cancer patients in Brazil. J Cancer Res Clin Oncol.

[29] Lee H., Hanson H.A., Logan J., Maguire D., Kapadia A., Dewji S. (2024). Evaluating county-level lung cancer incidence from environmental radiation exposure, PM2.5, and other exposures with regression and machine learning models. Env Geochem Health.

[30] Petousis P., Han S.X., Aberle D., Bui AA. (2016). Prediction of lung cancer incidence on the low-dose computed tomography arm of the national lung screening trial: a dynamic Bayesian network. Artif Intell Med.

[31] Calvo V., Niazmand E., Carcereny E., Rodríguez-Abreu D., Cobo M., López-Castro R. (2024). Family history of cancer and lung cancer: utility of big data and artificial intelligence for exploring the role of genetic risk. Lung Cancer.

[32] Leiter A., Charokopos A., Bailey S., Gallagher E.J., Hirsch F.R., LeRoith D. (2021). Assessing the association of diabetes with lung cancer risk. Transl Lung Cancer Res.

[33] Ma Z., Lv J., Zhu M., Yu C., Ma Y., Jin G. (2023). Lung cancer risk score for ever and never smokers in China. Cancer Commun.

[34] Huang S., Yang J., Shen N., Xu Q., Zhao Q. (2023). Artificial intelligence in lung cancer diagnosis and prognosis: current application and future perspective. Semin Cancer Biol.

