# Supplementary material for: Epidemiological trend comparison and incidence prediction for elderly lung cancer in 204 countries and territories: a retrospective study
Source: Clinics (Sao Paulo). 2026 Oct 15;81:101161. doi: 10.1016/j.clinsp.2026.101161 (PMC13691505; doi:10.1016/j.clinsp.2026.101161)
Supplement: Supplementary file 1 [file mmc1.docx]

Supplementary:

Supplementary Table 1. Age standardized mortality, proportion of disability adjusted life years (DALYs) and average annual percentage change (AAPC) of lung cancer in aged over 65 years of global and SDI levels from 1990 to 2021.

| Group | Mortality | | | | | DALYs | | | | |
| --- | --- | --- | --- | --- | --- | --- | --- | --- | --- | --- |
|  | Cases in 1990 (thousands) | Age standardized rate in 1990 (per 100,000) | Cases in 2021 (thousands) | Age standardized rate in 2021 (per 100,000) | AAPC 1990-2021 (%/year, 95% CI) | Cases in 1990 (thousands) | Age standardized rate in 1990 (per 100,000) | Cases in 2021 (thousands) | Age standardized rate in 2021 (per 100,000) | AAPC 1990-2021 (%/year, 95% CI) |
| Global  (age≥65) | 600(565-631) | 183(172-191) | 1386(1238-1519) | 174(153-189) | -0.26(-0.33-  -0.20) | 11375(10749-11945) | 3695(3502-3874) | 24462(21958-26787) | 3065(2748-3366) | -0.50(-0.56-  -0.44) |
| **Gender (age ≥65):** | | | | | | | | | | |
| Male | 430(406-456) | 306(288-324) | 909(795-1021) | 483(210-274) | -0.67(-0.73-  -0.60) | 8272(7794-8786) | 6237(5894-6609) | 16350(14319-18421) | 4541(3966-5134) | -0.87(-0.95-  -0.82) |
| Female | 170(155-181) | 94(86-100) | 477(410-532) | 101(89-113) | 0.44(0.38-0.50) | 3103(2855-3312) | 1739(1610-1851) | 8112(7080-9049) | 1822(1592-2038) | 0.21(0.15-0.27) |
| **SDI (age ≥65):** | | | | | | | | | | |
| High | 281(265-288) | 248(236-255) | 469(419-497) | 176(157-187) | -0.81(-0.90-  -0.71) | 5170(4937-5300) | 4873(4668-4995) | 7782(7085-8189) | 3203(2907-3383) | -1.12(-1.21-  -1.03) |
| High-middle | 177(165-189) | 212(198-226) | 439(382-498) | 207(178-238) | 0.21(0.12-0.29) | 3431(3197-3666) | 4272(3982-4562) | 7908(6913-9006) | 3877(3343-4485) | -0.06(-0.16-  0.04) |
| Middle | 111(99-122) | 150(136-164) | 391(325-452) | 158(131-183) | 0.46(0.38-0.54) | 2149(1927-2371) | 2980(2690-3273) | 7098(5916-8238) | 2967(2459-3467) | 0.26(0.18-0.34) |
| Low-middle | 24(21-28) | 58(51-67) | 72(65-79) | 63(57-69) | 0.37(0.35-0.40) | 481(420-561) | 1196(1056-1384) | 1368(1245-1509) | 1262(1151-1388) | 0.29(0.25-0.32) |
| Low | 6(5-8) | 42(36-52) | 14(12-17) | 41(36-48) | -0.13(-0.23-  -0.03) | 130(108-164) | 890(750-1101) | 281(242-327) | 839(733-968) | -0.25(-0.35-  -0.14) |
| **Age:** |  |  |  |  |  |  |  |  |  |  |
| 65-69 | 187(178-197) | 158(150-166) | 329(297-363) | 117(129-105) | -0.90(-0.95-  -0.84) | 4602(4360-4844) | 3879(3684-4075) | 8085(7292-8910) | 2875(2587-3174) | -0.89(-0.94-  -0.83) |
| 70-74 | 159(151-168) | 195(184-205) | 349(313-386) | 167(150-185) | -0.53(-0.64-  -0.41) | 3223(3051-3385) | 3963(3760-4154) | 7070(6335-7816) | 3378(3021-3741) | -0.52(-0.63-  -0.41) |
| 75-79 | 133(126-139) | 223(212-232) | 282(252-310) | 211(189-233) | -0.06(-0.20-  -0.08) | 2152(2037-2250) | 3652(3466-3812) | 4557(4085-5018) | 3399(3041-3748) | -0.06(-0.20-0.08) |
| 80-84 | 77(71-81) | 224(206-235) | 217(186-238) | 246(209-270) | 0.51(0.34-0.67) | 976(896-1028) | 2915(2689-3062) | 2746(2365-3018) | 3079(2644-3389) | 0.50(0.33-0.66) |
| 85-89 | 33(29-35) | 223(196-237) | 137(116-151) | 298(251-328) | 1.14(1.00-  1.29) | 330(291-350) | 2339(2079-2474) | 1381(1164-1526) | 2965(2489-3282) | 1.12(0.97-1.28) |
| 90-94 | 90(75-97) | 216(182-234) | 56(46-62) | 311(249-346) | 1.39(1.31-1.47) | 79(66-85) | 1997(1697-2146) | 490(391-548) | 2684(2126-3006) | 1.40(1.32-1.48) |
| ≥95 | 2(1-3) | 181(141-202) | 16(12-19) | 294(212-339) | 1.75(1.66-1.83) | 15(11-16) | 1598(1271-1764) | 131(95-151) | 2352(1682-2717) | 1.63(1.45-1.82) |

Supplementary Table 2. Age standardized incidence, mortality and DALYs of lung cancer in the male and female elderly at regional levels in 2021.

| Regions | Age standardized rate in 2021 (per 100,000) | | | | | |
| --- | --- | --- | --- | --- | --- | --- |
|  | Male | | | Female | | |
|  | Incidence | Mortality | DALYs | Incidence | Mortality | DALYs |
| Andean Latin America | 83.5(66.1-103.8) | 91.8(72.2-113.5) | 1675(1303-2098) | 59.2(43.6-75.9) | 64.9(47.7-83.3) | 1184(859-1532) |
| Australasia | 236(204-270) | 185(162-207) | 3339(2959-3723) | 152(121-177) | 111(90-128) | 2021(1656-2310) |
| Caribbean | 205(176-234) | 191(165-217) | 3599(3114-4104) | 83.9(70.4-97.5) | 88.8(75.1-103) | 1652(1389-1930) |
| Central Asia | 158(142-173) | 169(152-185) | 3463(3106-3801) | 30.6(27.3-33.9) | 33.3(29.7-37.1) | 639(569-711) |
| Central Europe | 347(315-377) | 354(323-383) | 7031(6376-7608) | 110(96.2-122) | 111(97.6-123) | 2185(1935-2419) |
| Central Latin America | 85.8(74.8-97.3) | 93.2(81.2-105) | 1750(1520-1992) | 46.4(39.4-53.5) | 50.9(43.2-58.6) | 933(793-1082) |
| Central Sub-Saharan Africa | 103(71.6-159) | 114(78.9-179) | 2349(1629-3686) | 29.8(22.3-40.5) | 33.1(24.6-45.6) | 675(503-928) |
| East Asia | 394(287-507) | 360(264-463) | 6597(4768-8598) | 169(129-213) | 153(118-193) | 2756(2116-3498) |
| Eastern Europe | 289(258-321) | 270(241-299) | 5481(4869-6094) | 42.2(36.6-47.4) | 38.8(33.9-42.9) | 726(634-817) |
| Eastern Sub-Saharan  Africa | 61.7(52.3-71.9) | 68.8(58.3-80.4) | 1371(1174-1606) | 25.3(20.5-30.1) | 28.2(22.2-33.1) | 576(465-683) |
| High-income Asia  Pacific | 321(277-350) | 239(208-258) | 4079(3633-4389) | 89.7(61.9-108) | 66.5(43.6-80.1) | 1039(751-1224) |
| High-income North America | 283(262-298) | 231(213-244) | 4234(3954-4443) | 199(169-216) | 154(131-167) | 2769(2407-2973) |
| North Africa and Middle East | 178(152-209) | 198(169-233) | 3836(3313-4258) | 43.4(37.3-51.5) | 47.6(40.1-56.2) | 893(745-1042) |
| Oceania | 169(126-246) | 190(139-276) | 3769(2763-5498) | 71.6(52.1-102) | 78.8(57.2-112) | 1606(1163-2296) |
| South Asia | 59.1(48.3-68.4) | 62.9(51.2-74.8) | 1322(1081-1535) | 20.5(17.5-23.84) | 22.8(19.2-26.1) | 445(379-517) |
| Southeast Asia | 204(169-237) | 224(185-255) | 4367(3612-5122) | 73.1(55.9-87.9) | 80(61.3-96.6) | 1533(1167-1852) |
| Southern Latin America | 205(174-216) | 206(185-228) | 3986(3580-4415) | 75.4(64.7-86.3) | 78.8(67.8-89.8) | 1486(1285-1702) |
| Southern Sub-Saharan Africa | 177(157-196) | 194(173-216) | 3905(3476-4371) | 69.2(59.4-79.2) | 76.3(65.9-87.4) | 1473(1264-1691) |
| Tropical Latin America | 129(120-137) | 141(129-149) | 2670(2472-2835) | 67.1(58.5-73.1) | 72.9(63.2-79.5) | 1381(1221-1507) |
| Western Europe | 276(249-295) | 241(219-257) | 4452(4099-4718) | 116(99.2-129) | 96.5(81.1-106) | 1782(1545-1938) |
| Western Sub-Saharan Africa | 46.5(39.3-54.7) | 51.8(43.9-61.1) | 1037(876-1230) | 14.4(11.6-16.8) | 15.9(12.8-18.6) | 318(256-372) |

Supplementary Table 3. Age standardized mortality, proportion of disability adjusted life years (DALYs) and average annual percentage change (AAPC) of lung cancer in aged over 65 years of the regional levels from 1990 to 2021.

| Regions | Mortality | | | | | DALYs | | | | |
| --- | --- | --- | --- | --- | --- | --- | --- | --- | --- | --- |
|  | Cases in 1990 (thousands) | Age standardized rate in 1990 (per 100,000) | Cases in 2021 (thousands) | Age standardized rate in 2021 (per 100,000) | AAPC 1990-2021 (%/year, 95% CI) | Cases in 1990 (thousands) | Age standardized rate in 1990 (per 100,000) | Cases in 2021 (thousands) | Age standardized rate in 2021 (per 100,000) | AAPC 1990-2021 (%/year, 95% CI) |
| Andean Latin America | 1.60(1.39-1.84) | 101(87.7-115) | 4.23(3.31-5.18) | 77.3(60.7-94.8) | -0.94(-1.33-  -0.75) | 29.0(25.0-33.6) | 1949(1693-2242) | 72.2(56.1-89.0) | 1414(1097-1743) | -1.20(-1.40-  -0.99) |
| Australasia | 5.34(4.96-5.65) | 224(208-237) | 9.56(8.33-10.7) | 145(125-163) | -1.45(-1.53-  -1.36) | 99.8(93.2-105.6) | 4436(4135-4709) | 160(141-176) | 2634(2313-2928) | -1.82(-1.89-  -1.75) |
| Caribbean | 3.64(3.42-3.88) | 155(146-165) | 7.06(6.24-7.93) | 136(119-153) | -0.10(-0.22-0.01) | 64.4(60.6-68.7) | 2932(2759-3129) | 123(109-139) | 2543(2227-2868) | -0.13(-0.25-  -0.01) |
| Central Asia | 4.75(4.50-4.99) | 148.2(140.7-155.2) | 5.22(4.70-5.73) | 87.8(79.3-96.3) | -1.38(-1.50-  -1.26) | 95.0(90.1-99.9) | 3175(3023-3326) | 104(94.1-115) | 1783(1608-1957) | -1.66(-1.77-  -1.54) |
| Central Europe | 27.5(26.3-28.5) | 201(193-209) | 55.1(51.0-58.8) | 209(190-225) | 0.06(-0.03-0.16) | 540(520-560) | 4051(3893-4210) | 1041(963-1109) | 4163(3796-4485) | -0.08(-0.19-  0.03) |
| Central Latin America | 6.95(6.62-7.17) | 110(105-113) | 16.0(14.1-18.0) | 69.8(61.2-78.8) | -1.68(-1.77-  -1.58) | 126(121-130) | 2119(2040-2183) | 282(249-318) | 1302(1149-1474) | -1.84(-1.94-  -1.73) |
| Central Sub-Saharan Africa | 1.01(0.71-1.53) | 71.6(52.1-106) | 2.15(1.55-3.20) | 66.8(49.7-96.6) | -0.17(-0.43-0.09) | 20.8(14.5-31.8) | 1516(1099-2243) | 43.5(31.3-65.1) | 1384(1031-2011) | -0.24(-0.50-0.01) |
| East Asia | 147(128-167) | 223(195-253) | 579(471-692) | 248(198-302) | 0.44(0.21-  0.68) | 2865(2475-3265) | 4456(3866-5063) | 10356(8402-12456) | 4530(3583-5556) | 0.08(-0.13-  0.29) |
| Eastern Europe | 40.9(39.6-42.1) | 169(163-174) | 44.0(40.4-47.6) | 116(105-127) | -1.42(-1.54-  -1.31) | 816(790-838) | 3502(3385-3602) | 859(788-932) | 2340(2106-2575) | -1.64(-1.78-  -1.49) |
| Eastern Sub-Saharan  Africa | 2.55(2.15-3.04) | 51.4(43.9-60.4) | 5.17(4.47-6.03) | 46.5(41.1-53.8) | -0.39(-0.48-  -0.30) | 52.2(44.1-62.6) | 1088(936-1286) | 100(86.7-118) | 943(826-1092) | -0.57(-0.66-  -0.48) |
| High-income Asia  Pacific | 35.2(32.9-36.7) | 181(169-189) | 104(87.3-113) | 139(113-156) | -0.92(-1.02-  -0.83) | 620(585-644) | 3344(3158-3478) | 1539(1332-1660) | 2361(2002-2593) | -1.22(-1.32-  -1.13) |
| High-income North America | 115(107-119) | 310(292-322) | 153(138-162) | 189(168-201) | -1.76(-2.05-  -1.47) | 2138(2024-2209) | 6232(5927-6432) | 2595(2359-2732) | 3425(3102-3617) | -2.15(-2.42-  -1,88) |
| North Africa and Middle East | 14.5(11.9-17.5) | 124(104-148) | 41.5(35.9-48.6) | 122(106-142) | 0.05(-0.07-0.17) | 282(231-341) | 2520(2106-3006) | 779(674-915) | 2373(2060-2772) | -0.17(-0.28-  -0.06) |
| Oceania | 0.24(0.17-0.34) | 129(97-182) | 0.64(0.47-0.92) | 136(103-192) | 0.20(0.15-  0.24) | 4.77(3.47-6.92) | 2645(1982-3738) | 12.4(9.19-17.9) | 2724(2059-3837) | 0.10(0.04-0.17) |
| South Asia | 15.3(13.1-17.8) | 40.4(35.9-46.7) | 50.8(42.9-57.7) | 42.8(36.3-48.6) | -0.01(-0.12-0.12) | 307(263-357) | 843(732-971) | 986(830-1123) | 866(732-985) | -0.11(-0.24-0.01) |
| Southeast Asia | 24.5(21.2-27.7) | 139(121-156) | 74.8(61.1-86.3) | 143(117-165) | -0.11(-0.22-0.01) | 477(414-541) | 2822(2466-3171) | 1413(1154-1639) | 2768(2262-3215) | -0.30(-0.42-  -0.18) |
| Southern Latin America | 7.21(6.69-7.74) | 173(160-185) | 11.9(10.7-13.1) | 132(119-146) | -0.64(-0.76-  -0.53) | 136(127-146) | 3458(3212-3710) | 213(193-234) | 2556(2309-2821) | -0.79(-0.90-  -0.68) |
| Southern Sub-Saharan Africa | 2.10(1.82-2.52) | 104(91.3-124) | 5.37(4.86-5.96) | 122(111-135) | 0.48(0.17-0.80) | 39.5(34.1-47.7) | 2095(1834-2498) | 103(92.9-114) | 2423(2202-2683) | 0.42(0.09-0.75) |
| Tropical Latin America | 7.63(7.18-7.99) | 110(104-115) | 24.5(22.1-26.2) | 102(92.1-109) | -0.24(-0.34-  -0.14) | 145(138-152) | 2216(2112-2310) | 442(404-470) | 1938(1776-2066) | -0.48(-0.58-  -0.37) |
| Western Europe | 136(129-140) | 217(206-223) | 189(168-201) | 159(142-171) | -0.88(-0.95-  -0.82) | 2483(2378-2550) | 4233(4056-4353) | 3157(2870-3335) | 2968(2695-3152) | -1.10(-1.17-  -1.03) |
| Western Sub-Saharan Africa | 1.68(1.40-1.99) | 26.2(22.3-30.8) | 4.28(3.65-5.04) | 32.8(28.3-38.4) | 0.97(0.89-1.05) | 32.6(27.2-38.7) | 534(453-626) | 81.9(69.0-97.3) | 646(561-773) | 0.89(0.82-0.97) |

Supplementary Table 4. Age standardized incidence, mortality and DALYs of lung cancer in aged over 65 years elderly in 2021 and their average annual percentage change (AAPC) between 1990-2021 in 204 countries and territories.

| Countries and territories | Age standardized rate in 2021 (per 100,000) | | | APPC between 1990-2021 (%/year) | | |
| --- | --- | --- | --- | --- | --- | --- |
|  | Incidence | Mortality | DALYs | Incidence | Mortality | DALYs |
| Afghanistan | 50.1(30.5-81.3) | 70.8(48.8-106) | 1412(972-2076) | 0.81(0.71-0.93) | 0.83(0.72-0.94) | 0.74(0.65-0.83) |
| Albania | 139(92.7-195) | 147(97.6-207) | 2740(1813-3842) | -0.79(-1.02-  -0.58) | -0.91(-1.13-  -0.68) | -1.22(-1.43-  -0.99) |
| Algeria | 41.3(29.1-57.1) | 46.5(33.1-64.4) | 881(619-1220) | -0.55(-0.73-  -0.36) | -0.53(-0.72-  -0.33) | -0.73(-0.90-  -0.56) |
| American Samoa | 169(135-213) | 188(151-236) | 3598(2842-4521) | -0.38(-0.43-  -0.32) | -0.37(-0.43-  -0.31) | -0.53(-0.59-  -0.47) |
| Andorra | 162(99.5-232) | 145(92.8-205) | 2638(1629-3810) | -1.60(-1.98-  -1.22) | -1.89(-2.28-  -1.51) | -2.15(-2.54-  -1.75) |
| Angola | 78.7(58.9-101) | 86.9(64.8-112) | 1800(1337-2336) | -0.07(-0.19-  -0.05) | -0.06(-0.18-0.07) | -0.16(-0.28-  -0.04) |
| Antigua and Barbuda | 64.1(56.2-73.8) | 64.1(56.7-72.9) | 1227(1073-1409) | -0.29(-0.62-  0.03) | -0.45(-0.76-  -0.13) | -0.61(-0.92-  -0.28) |
| Argentina | 131(116-145) | 141(126-156) | 2721(2434-3022) | -0.47(-0.60-  -0.33) | -0.49(-0.61-  -0.37) | -0.66(-0.79-  -0.52) |
| Armenia | 163(143-185) | 173(152-196) | 3403(2978-3876) | -0.12(-0.51-  0.28) | -0.11(-0.49-  0.27) | -0.52(-0.92-  -0.12) |
| Australia | 190(161-217) | 144(123-162) | 2607(2281-2913) | -0.78(-0.91-  -0.65) | -1.43(-1.53-  -1.34) | -1.82(-1.89-  -1.74) |
| Austria | 165(143-188) | 141(123-158) | 2705(2390-3016) | 0.23(0.09-  0.38) | -0.32(-0.48-  -0.16) | -0.32(-0.51-  -0.13) |
| Azerbaijan | 83.6(58.9-114) | 90.1(63.3-122) | 1832(1255-2503) | -0.31(-0.58-  -0.05) | -0.30(-0.57-  -0.04) | -0.61(-0.86-  -0.37) |
| Bahamas | 82.9(66.8-102) | 105(88.6-125) | 1648(1320-2024) | -0.30(-0.46-  -0.15) | 0.29(0.13-0.46) | -0.63(-0.83-  -0.42) |
| Bahrain | 140(112-181) | 155(124-201) | 2906(2292-3838) | -2.80(-3.15-  -2.46) | -2.81(-3.16-  -2.46) | -3.12(-3.49-  -2.76) |
| Bangladesh | 32.4(23.6-44.7) | 35.8(25.9-48.9) | 706(508-977) | -1.28(-1.49-  -1.07) | -1.30(-1.52-  -1.09) | -1.46(-1.68-  -1.26) |
| Barbados | 53.9(40.7-68.9) | 54.9(41.6-70.3) | 994(738-1296) | -0.26(-0.45-  -0.07) | -0.39(-0.57-  -0.21) | -0.54(-0.72-  -0.36) |
| Belarus | 118(89.9-147) | 113(86.1-139) | 2279(1708-2868) | -1.42(-1.76-  -1.08) | -1.73(-2.08-  -1.38) | -1.89(-2.29-  -1.49) |
| Belgium | 210(179-239) | 189(162-215) | 3576(3113-4012) | -1.01(-1.22-  -0.80) | -1.37(-1.58-  -1.15) | -1.54(-1.73-  -1.35) |
| Belize | 71.3(61.4-82.0) | 75.2(64.8-86.3) | 1448(1239-1668) | 0.16(-0.43-  0.75) | 0.05(-0.54-  0.65) | -0.05(-0.66-  0.57) |
| Benin | 45.9(35.5-60.2) | 51.3(39.6-67.6) | 1023(785-1356) | 0.34(0.18-0.51) | 0.35(0.19-0.51) | 0.30(0.14-0.47) |
| Bermuda | 157(123-203) | 128(103-164) | 2256(1790-2904) | -1.24(-1.43-  -1.05) | -2.03(-2.19-  -1.86) | -2.31(-2.47-  -2.15) |
| Bhutan | 33.9(22.6-48.3) | 37.6(25.3-53.8) | 728(482-1053) | 0.57(0.44-0.69) | 0.60(0.48-0.73) | 0.27(0.15-0.39) |
| Bolivia (Plurinational State of) | 92.8(65.6-130) | 104(73.2-146) | 1975(1371-2798) | -0.32(-0.40-  -0.24) | -0.31(-0.39-  -0.23) | -0.53(-0.61-  -0.44) |
| Bosnia and Herzegovina | 190(135-254) | 198(142-266) | 3873(2743-5185) | 0.18(0.01-0.35) | 0.06(-0.10-0.21) | -0.15(-0.33-  0.02) |
| Botswana | 99.4(71.4-130) | 110(79.8-143) | 2205(1593-2910) | -0.38(-0.67-  -0.08) | -0.35(-0.65-  -0.06) | -0.54(-0.86-  -0.22) |
| Brazil | 93.4(84.8-99.9) | 102(91.9-109) | 1930(1770-2059) | -0.26(-0.36-  -0.16) | -0.28(-0.38-  -0.19) | -0.52(-0.62-  -0.41) |
| Brunei Darussalam | 183(144-222) | 188(149-228) | 3461(2728-4244) | -1.19(-1.38-  -1.02) | -1.34(-1.53-  -1.15) | -1.54(-1.71-  -1.37) |
| Bulgaria | 161(128-196) | 161(130-193) | 3292(2646-3946) | 1.26(1.08-1.43) | 1.10(0.94-1.28) | 1.07(0.91-1.23) |
| Burkina Faso | 42.1(30.7-61.3) | 47.0(34.6-67.6) | 943(688-1360) | 0.85(0.68-1.03) | 0.87(0.70-1.04) | 0.79(0.61-0.98) |
| Burundi | 38.0(26.9-54.1) | 42.2(29.8-60.5) | 873(612-1256) | -1.12(-1.34-  -0.89) | -1.08(-1.29-  -0.85) | -1.19(-1.43-  -0.97) |
| Cabo Verde | 126(92.9-157) | 142(105-177) | 2536(1852-3203) | 1.30(0.86-1.73) | 1.28(0.83-1.72) | 1.08(0.65-1.52) |
| Cambodia | 162(116-217) | 178(130-239) | 3583(2563-4815) | 0.05(-0.01-  0.10) | 0.05(-0.01-0.11) | -0.14(-0.19-  -0.09) |
| Cameroon | 58.6(42.7-77.6) | 65.1(47.1-85.8) | 1315(925-1749) | 0.71(0.60-0.81) | 0.69(0.59-0.81) | 0.68(0.56-0.78) |
| Canada | 236(205-266) | 190(167-211) | 3391(3017-3729) | -0.88(-1.17-  -0.59) | -1.35(-1.56-  -1.14) | -1.77(-1.96-  -1.56) |
| Central African Republic | 60.5(40.1-105) | 66.6(44.2-116) | 1426(944-2469) | -0.61(-0.66-  -0.57) | -0.59(-0.64-  -0.55) | -0.65(-0.70-  -0.61) |
| Chad | 52.5(37.5-75.2) | 58.5(41.6-83.2) | 1191(845-1692) | 1.87(1.74-2.01) | 1.86(1.73-2.01) | 1.87(1.73-2.02) |
| Chile | 100(87.6-112) | 102(89.3-113) | 1885(1672-2095) | -0.14(-0.28-  0.01) | -0.38(-0.52-  -0.24) | -0.62(-0.76-  -0.48) |
| China | 276(219-336) | 251(199-306) | 4586(3601-5641) | 1.07(0.87-1.28) | 0.46(0.23-0.70) | 0.14(-0.09-  0.37) |
| Colombia | 74.7(61.1-89.7) | 80.1(65.9-96.3) | 1478(1213-1787) | -1.41(-1.52-  -1.31) | -1.50(-1.60-  -1.40) | -1.77(-1.88-  -1.65) |
| Comoros | 47.4(30.9-66.7) | 52.8(34.7-74.8) | 1053(694-1489) | -0.12(-0.20-  -0.04) | -0.08(-0.16-0.01) | -0.28(-0.36  -0.19) |
| Congo | 84.8(62.9-112) | 93.9(70.1-124) | 1907(1410-2543) | -0.36(-0.50-  -0.22) | -0.34(-0.49-  -0.21) | -0.49(-0.62-  -0.36) |
| Cook Islands | 174(133-217) | 187(143-234) | 3428(2612-4336) | -1.19(-1.31-  -1.08) | -1.31(-1.43-  -1.19) | -1.51(-1.63-  -1.40) |
| Costa Rica | 52.9(45.1-61.0) | 55.8(47.3-64.4) | 1028(875-1191) | -1.38(-1.61-  -1.15) | -1.46(-1.69-  -1.22) | -1.68(-1.91-  -1.46) |
| Croatia | 224(188-260) | 189(160-219) | 3733(3155-4318) | -0.04(-0.22-  0.14) | -0.52(-0.69-  -0.35) | -0.63(-0.83-  -0.41) |
| Cuba | 221(184-261) | 209(176-243) | 3936(3308-4594) | 0.44(0.28-0.60) | 0.12(-0.03-0.28) | 0.15(0.01-0.32) |
| Cyprus | 146(113-186) | 132(102-166) | 2493(1913-3123) | 0.53(0.24-0.83) | -0.10(-0.35-0.15) | -0.14(-0.39-  0.11) |
| Czechia | 199(166-235) | 179(152-209) | 3558(3007-4167) | -0.62(-0.72-  -0.51) | -1.09(-1.16-  -1.01) | -1.19(-1.27-  -1.12) |
| Côte d'Ivoire | 28.2(20.9-38.1) | 28.8(21.4-39.3) | 637(466-877) | -0.36(-0.52-  -0.21) | -0.37(-0.53-  -0.22) | -0.45(-0.61-  -0.29) |
| Democratic People's Republic of Korea | 132(83.4-193) | 144(90.8-212) | 2829(1759-4169) | 0.01(-0.17-  0.20) | -0.07(-0.25-0.10) | -0.11(-0.32-  0.10) |
| Democratic Republic of the Congo | 50.9(33.1-87.5) | 56.3(36.4-98.1) | 1170(756-2045) | -0.35(-0.71-  0.01) | -0.33(-0.69-0.03) | -0.38(-0.73-  -0.02) |
| Denmark | 252(213-290) | 224(191-256) | 4144(3604-4696) | -0.47(-0.65-  -0.29) | -0.99(-1.10-  -0.87) | -1.34(-1.42-  -1.25) |
| Djibouti | 68.4(45.2-96.2) | 75.5(49.8-106) | 1552(1022-2182) | 0.73(0.70-0.75) | 0.74(0.72-0.77) | 0.63(0.60-0.66) |
| Dominica | 112(86.5-143) | 121(92.8-152) | 2288(1741-2921) | -0.07(-0.24-  0.09) | -0.11(-0.27-  0.05) | -0.20(-0.41-  0.01) |
| Dominican Republic | 82.8(54.9-115) | 89.6(59.5-125) | 1677(1098-2357) | 1.10(0.92-1.29) | 1.02(0.83-1.21) | 0.94(0.79-1.08) |
| Ecuador | 52.8(41.8-65.8) | 59.2(47.3-73.4) | 1044(823-1310) | -0.43(-0.76-  -0.11) | -0.44(-0.78-  -0.09) | -0.74(-1.06-  -0.43) |
| Egypt | 89.1(70.2-111) | 99.3(78.4-124) | 1971(1542-2484) | 4.62(3.99-5.25) | 4.65(4.01-5.29) | 4.44(3.83-5.05) |
| El Salvador | 56.6(44.8-71.7) | 62.1(48.9-79.3) | 1151(907-1473) | -0.13(-0.30-  0.04) | -0.19(-0.35-  -0.03) | -0.42(-0.59-  -0.24) |
| Equatorial Guinea | 95.9(64.7-135) | 105(71.0-147) | 2123(1425-2975) | 1.26(1.16-1.35) | 1.25(1.15-1.34) | 1.02(0.93-1.12) |
| Eritrea | 51.6(39.6-65.3) | 56.9(43.5-71.8) | 1189(901-1505) | 0.41(0.23-0.60) | 0.45(0.26-0.63) | 0.28(0.09-0.47) |
| Estonia | 148(120-176) | 151(123-178) | 2955(2403-3473) | -0.78(-0.93-  -0.63) | -0.99(-1.13-  -0.85) | -1.20(-1.36-  -1.04) |
| Eswatini | 120(73.3-171) | 131(80.2-186) | 2756(1697-3910) | 0.67(0.25-1.11) | 0.65(0.22-1.08) | 0.66(0.21-1.12) |
| Ethiopia | 46.9(38.2-56.7) | 52.6(43.1-63.7) | 1036(848-1251) | -1.64(-1.90-  -1.38) | -1.56(-1.82-  -1.31) | -1.89(-2.14-  -1.64) |
| Fiji | 62.4(45.9-81.2) | 69.5(51.1-89.3) | 1362(988-1782) | -0.68(-0.88-  -0.47) | -0.69(-0.90-  -0.48) | -0.75(-0.94-  -0.56) |
| Finland | 155(129-181) | 123(104-141) | 2326(1991-2669) | -0.80(-0.94-  -0.65) | -1.30(-1.43-  -1.17) | -1.51(-1.68-  -1.34) |
| France | 188(161-215) | 153(132-173) | 2917(2551-3260) | 0.63(0.49-0.76) | -0.21(-0.31-  -0.10) | -0.39(-0.49-  -0.28) |
| Gabon | 102(72.9-136) | 112(80.5-149) | 2271(1608-3019) | 0.21(0.16-0.26) | 0.19(0.14-0.23) | 0.13(0.08-0.17) |
| Gambia | 21.7(15.8-29.5) | 23.9(17.3-32.4) | 489(354-659) | 0.10(-0.03-  0.02) | 0.10(-0.02-  0.23) | 0.02(-0.12-  0.15) |
| Georgia | 129(110-149) | 138(118-160) | 2796(2382-3265) | 1.49(1.05-1.94) | 1.54(1.08-2.01) | 1.34(0.92-1.76) |
| Germany | 186(161-212) | 156(136-176) | 3001(2646-3337) | -0.06(-0.23-  0.11) | -0.52(-0.66-  -0.38) | -0.60(-0.75-  -0.45) |
| Ghana | 44.1(32.9-57.0) | 49.2(36.8-63.3) | 966(720-1265) | 0.92(0.80-1.03) | 0.90(0.78-1.02) | 0.86(0.74-0.99) |
| Greece | 234(203-262) | 218(191-242) | 4177(3702-4626) | -0.47(-0.57-  -0.37) | -0.70(-0.83-  -0.57) | -0.64(-0.77-  -0.51) |
| Greenland | 403(307-519) | 422(321-539) | 8196(6185-10468) | -1.71(-1.87-  -1.55) | -1.84(-1.99-  -1.68) | -1.99(-2.17-  -1.82) |
| Grenada | 72.2(60.6-84.9) | 75.3(63.4-88.1) | 1457(1216-1716) | -0.45(-1.06-  0.17) | -0.56(-1.18-  0.07) | -0.68(-1.21-  -0.16) |
| Guam | 135(105-166) | 145(113-178) | 2889(2261-3576) | -1.62(-1.95-  -1.29) | -1.68(-2.02-  -1.33) | -1.76(-2.12-  -1.40) |
| Guatemala | 36.5(31.1-42.4) | 41.2(34.9-47.9) | 760(641-887) | -2.06(-2.36-  -1.76) | -2.07(-2.36-  -1.77) | -2.25(-2.53-  -1.96) |
| Guinea | 53.9(38.2-73.1) | 60.3(42.8-83.1) | 1210(862-1685) | 1.05(0.96-1.15) | 1.06(0.97-1.15) | 1.04(0.95-1.13) |
| Guinea-Bissau | 59.8(45.1-78.9) | 66.2(50.1-87.5) | 1375(1036-1823) | 0.36(0.16-0.55) | 0.36(0.16-0.55) | 0.32(0.13-0.52) |
| Guyana | 41.6(32.2-52.6) | 45.3(35.1-56.9) | 903(690-1146) | -0.10(-0.29-  0.09) | -0.13(-0.32-0.07) | -0.21(-0.41-  -0.01) |
| Haiti | 72.5(47.1-115) | 80.3(51.1-126) | 1610(1018-2536) | -0.44(-0.52-  -0.36) | -0.43(-0.51-  -0.35) | -0.57(-0.65-  -0.49) |
| Honduras | 137(93.6-193) | 152(103-216) | 2999(2051-4290) | 2.01(1.80-2.21) | 1.97(1.76-2.17) | 1.94(1.74-2.14) |
| Hungary | 241(199-284) | 240(199-281) | 4882(4056-5710) | 0.22(0.10-0.34) | 0.02(-0.09-  0.13) | 0.05(-0.08-0.19) |
| Iceland | 210(173-245) | 168(140-193) | 3047(2575-3501) | -0.03(-0.39-  0.34) | -0.48(-0.79-  -0.17) | -0.79(-1.09-  -0.50) |
| India | 35.6(29.4-41.0) | 38.8(32.0-44.7) | 785(646-906) | 0.45(0.29-0.62) | 0.43(0.28-0.59) | 0.27(0.10-0.43) |
| Indonesia | 143(97.2-184) | 158(108-204) | 3134(2109-4049) | 1.02(0.95-1.10) | 1.01(0.93-1.09) | 0.87(0.80-0.95) |
| Iran (Islamic Republic of) | 63.9(57.0-70.6) | 73.1(65.1-80.6) | 1321(1186-1458) | 0.20(0.07-0.33) | 0.28(0.15-0.42) | -0.14(-0.26-  -0.02) |
| Iraq | 130(95.9-167) | 145(106-185) | 2842(2051-3683) | 0.90(0.69-1.10) | 0.88(0.67-1.09) | 0.74(0.56-0.93) |
| Ireland | 187(156-216) | 162(136-186) | 2965(2529-3385) | -0.84(-1.03-  -0.65) | -1.43(-1.61-  -1.23) | -1.71(-1.88-  -1.55) |
| Israel | 132(113-149) | 126(108-141) | 2304(2016-2573) | -0.39(-0.61-  -0.18) | -0.72(-0.91-  -0.52) | -0.98(-1.19-  -0.78) |
| Italy | 164(140-182) | 147(127-161) | 2678(2377-2874) | -0.94(-1.06-  -0.82) | -1.29(-1.37-  -1.22) | -1.64(-1.73-  -1.56) |
| Jamaica | 102(76.9-132) | 105(79.8-136) | 1982(1504-2581) | -0.02(-0.47-  0.43) | -0.11(-0.53-  0.31) | -0.35(-0.79-  0.10) |
| Japan | 177(144-198) | 129(105-143) | 2226(1902-2398) | -0.48(-0.58-  -0.37) | -1.02(-1.09-  -0.95) | -1.24(-1.31-  -1.16) |
| Jordan | 67.7(52.2-89.1) | 74.6(57.4-99.2) | 1465(1112-1976) | -0.31(-0.61-  -0.01) | -0.35(-0.64-  -0.06) | -0.53(-0.84-  -0.21) |
| Kazakhstan | 93.6(79.7-109) | 98.9(84.6-115) | 2052(1753-2383) | -2.54(-2.78-  -2.30) | -2.59(-2.85-  -2.33) | -2.77(-3.03-  -2.51) |
| Kenya | 22.7(18.1-28.3) | 24.9(19.9-31.1) | 517(414-646) | 1.78(1.62-1.94) | 1.77(1.60-1.93) | 1.74(1.56-1.91) |
| Kiribati | 102(76.7-137) | 114(86.2-154) | 2287(1715-3088) | 0.29(0.16-0.41) | 0.31(0.19-0.43) | 0.20(0.08-0.32) |
| Kuwait | 58.9(47.4-72.6) | 65.2(52.9-80.3) | 1183(956-1462) | -1.04(-1.58-  -0.49) | -1.02(-1.55-  -0.49) | -1.40(-1.87-  -0.93) |
| Kyrgyzstan | 70.1(58.1-83.7) | 74.9(61.8-89.9) | 1565(1287-1873) | -1.63(-1.96-  -1.29) | -1.63(-1.97-  -1.30) | -1.83(-2.14-  -1.51) |
| Lao People's Democratic Republic | 145(103-198) | 161(115-222) | 3215(2263-4483) | -0.37(-0.45-  -0.30) | -0.34(-0.41-  -0.26) | -0.56(-0.63-  -0.48) |
| Latvia | 142(114-171) | 133(107-158) | 2649(2134-3169) | -0.56(-0.74-  -0.37) | -0.83(-1.02-  -0.65) | -0.96(-1.16-  -0.76) |
| Lebanon | 148(111-195) | 165(124-218) | 3002(2242-3961) | 0.58(0.21-0.95) | 0.57(0.20-0.94) | 0.25(-0.11-  0.61) |
| Lesotho | 129(91.2-174) | 141(101-194) | 3026(2138-4182) | 2.40(2.23-2.56) | 2.36(2.20-2.53) | 2.48(2.31-2.65) |
| Liberia | 39.4(26.3-59.3) | 44.1(29.2-65.8) | 871(574-1312) | 0.41(0.13-0.70) | 0.42(0.14-0.69) | 0.34(0.05-0.63) |
| Libya | 157(112-215) | 176(126-243) | 3372(2398-4696) | 0.25(-0.03-0.52) | 0.25(-0.02-0.52) | 0.09(-0.19-  0.38) |
| Lithuania | 146(120-173) | 135(112-159) | 2690(2212-3173) | -0.78(-0.90-  -0.66) | -1.03(-1.15-  -0.92) | -1.13(-1.27-  -0.99) |
| Luxembourg | 191(163-220) | 172(149-195) | 3173(2759-3590) | -0.60(-0.78-  -0.42) | -1.05(-1.22-  -0.89) | -1.32(-1.48-  -1.16) |
| Madagascar | 35.4(26.2-45.3) | 39.1(28.7-49.8) | 812(590-1037) | -0.31(-0.44-  -0.19) | -0.31(-0.43-  -0.18) | -0.36(-0.48-  -0.23) |
| Malawi | 16.9(12.7-22.4) | 18.5(13.9-24.5) | 391(293-519) | 0.29(0.10-0.49) | 0.28(0.08-0.47) | 0.27(0.06-0.47) |
| Malaysia | 113(90.7-138) | 123(98.8-150) | 2383(1908-2922) | -0.20(-0.54-  0.14) | -0.29(-0.63-  0.06) | -0.42(-0.74-  -0.09) |
| Maldives | 59.8(45.9-74.8) | 66.7(51.5-83.6) | 1143(872-1455) | -2.36(-2.53-  -2.18) | -2.36(-2.55-  -2.19) | -2.94(-3.14-  -2.75) |
| Mali | 33.7(24.2-45.3) | 37.3(26.9-49.9) | 762(544-1030) | 0.71(0.56-0.85) | 0.70(0.55-0.85) | 0.65(0.51-0.79) |
| Malta | 108(88.5-127) | 102(83.6-121) | 1931(1599-2287) | -1.65(-1.89-  -1.41) | -1.96(-2.17-  -1.75) | -2.19(-2.40-  -1.96) |
| Marshall Islands | 150(86.7-227) | 205(137-289) | 4153(2713-5902) | 0.31(0.15-0.48) | 0.59(0.43-0.75) | 0.53(0.37-0.68) |
| Mauritania | 53.8(38.8-73.2) | 60.0(43.4-82.0) | 1167(836-1602) | 0.42(0.15-0.69) | 0.44(0.18-0.71) | 0.25(-0.02-0.52) |
| Mauritius | 59.9(52.7-66.8) | 65.4(57.8-72.6) | 1243(1093-1387) | -1.69(-1.99-  -1.38) | -1.70(-1.99-  -1.39) | -1.96(-2.25-  -1.67) |
| Mexico | 49.6(43.8-55.5) | 54.6(48.6-61.1) | 1001(886-1123) | -2.67(-2.85-  -2.49) | -2.72(-2.88-  -2.56) | -2.86(-3.04-  -2.67) |
| Micronesia (Federated States of) | 190(134-266) | 211(151-294) | 4238(2980-5941) | 0.21(0.17-0.25) | 0.21(0.17-0.25) | 0.09(0.05-0.13) |
| Monaco | 377(276-492) | 337(247-436) | 6206(4602-8039) | 1.37(0.92-1.83) | 1.07(0.62-1.53) | 0.93(0.49-1.36) |
| Mongolia | 149(115-192) | 165(128-212) | 3276(2567-4195) | -1.79(-2.03-  -1.55) | -1.79(-2.03-  -1.55) | -2.00(-2.26-  -1.75) |
| Montenegro | 279(205-369) | 282(208-371) | 5458(4009-7209) | 0.46(0.35-0.57) | 0.39(0.27-0.51) | 0.19(0.03-0.35) |
| Morocco | 89.2(62.9-115.7) | 99.8(71.1-129) | 1979(1397-2596) | 0.38(0.18-0.58) | 0.37(0.18-0.57) | 0.30(0.10-0.49) |
| Mozambique | 48.1(35.5-63.3) | 53.8(39.7-70.6) | 1083(802-1423) | 1.29(1.13-1.45) | 1.27(1.12-1.43) | 1.30(1.14-1.47) |
| Myanmar | 119(89.2-157) | 133(99.6-174.3) | 2603(1921-3473) | -0.71(-0.76-  -0.66) | -0.68(-0.73-  -0.64) | -0.93(-0.99  -0.87) |
| Namibia | 36.4(27.8-46.9) | 39.8(30.4-50.9) | 823(623-1061) | 0.49(0.29-0.68) | 0.47(0.26-0.67) | 0.40(0.19-0.60) |
| Nauru | 255(151-336) | 281(166-367) | 5751(3406-7568) | -0.16(-0.18-  -0.13) | -0.17(-0.19-  -0.15) | -0.21(-0.23-  -0.19) |
| Nepal | 35.2(24.9-48.9) | 38.9(27.6-54.3) | 776(545-1057) | 0.13(-0.13-  0.38) | 0.14(-0.11-  0.39) | -0.04(-0.30-  0.21) |
| Netherlands | 220(191-247) | 199(173-221) | 3708(3276-4088) | -0.83(-0.95-  -0.70) | -1.26(-1.36-  -1.16) | -1.51(-1.60-  -1.43) |
| New Zealand | 192(166-216) | 151(131-168) | 2272(2435-3083) | -0.89(-0.99-  -0.79) | -1.51(-1.61-  -1.40) | -1.82(-1.91-  -1.73) |
| Nicaragua | 33.7(26.6-41.9) | 36.8(28.8-45.9) | 705(552-881) | -0.38(-0.65-  -0.10) | -0.45(-0.73-  -0.16) | -0.55(-0.80-  -0.29) |
| Niger | 31.9(22.5-51.3) | 35.7(25.3-57.6) | 718(505-1163) | 0.55(0.37-0.74) | 0.58(0.39-0.76) | 0.48(0.28-0.67) |
| Nigeria | 10.5(8.68-12.7) | 11.7(9.76-14.2) | 230(191-282) | 0.83(0.71-0.94) | 0.85(0.73-0.96) | 0.68(0.58-0.79) |
| Niue | 179(126-240) | 199(142-266) | 3736(2607-5071) | 0.62(0.54-0.69) | 0.58(0.51-0.65) | 0.44(0.37-0.52) |
| North Macedonia | 169(126-218) | 176(132-226) | 3503(2575-4542) | 0.28(-0.01-  0.56) | 0.15(-0.12-  0.42) | 0.02(-0.27-  0.30) |
| Northern Mariana Islands | 242(197-288) | 263(214-311) | 5099(4089-6098) | -1.07(-1.17-  -0.97) | -1.09(-1.18-  -0.98) | -1.24(-1.31-  -1.17) |
| Norway | 210(182-232) | 162(143-175) | 2995(2676-3209) | 0.98(0.72-1.24) | 0.32(0.10-0.55) | -0.03(-0.23-  0.17) |
| Oman | 32.5(24.3-42.8) | 35.6(26.7-47.2) | 705(526-939) | -0.45(-0.72-  -0.17) | -0.47(-0.75-  -0.19) | -0.66(-0.95-  -0.36) |
| Pakistan | 89.8(67.9-116) | 99.3(75.2-129) | 2025(1535-2665) | 0.06(-0.19-  0.32) | 0.05(-0.21-  0.31) | 0.03(-0.23-0.28) |
| Palau | 285(219-365) | 316(243-401) | 5978(4518-7733) | -0.17(-0.28-  -0.05) | -0.18(-0.30-  -0.05) | -0.37(-0.46-  -0.28) |
| Palestine | 122(98.9-148) | 136(110-164) | 2653(2136-3196) | -0.41(-0.62-  -0.20) | -0.44(-0.66-  -0.23) | -0.47(-0.67-  -0.26) |
| Panama | 54.9(41.9-67.2) | 59.6(44.9-72.8) | 1081(821-1331) | -1.76(-1.94-  -1.57) | -1.78（-1.97-  -1.58） | -2.06(-2.24-  -1.89) |
| Papua New Guinea | 128(85.6-201) | 142(94.8-223) | 2856(1899-4476) | 0.53(0.51-0.56) | 0.56(0.53-0.59) | 0.46(0.42-0.50) |
| Paraguay | 106(79.3-142) | 116(86.1-156) | 2230(1649-3018) | 1.71(1.45-1.97) | 1.69(1.43-1.95) | 1.52(1.26-1.78) |
| Peru | 73.4(52.6-95.6) | 79.8(57.1-104) | 1449(1028-1894) | -1.18(-1.55-  -0.80) | -1.28(-1.64-  -0.92) | -1.54(-1.92-  -1.16) |
| Philippines | 106(89.1-125) | 117(98.6-138) | 2359(1965-2793) | -0.27(-0.43-  -0.11) | -0.33(-0.49-  -0.16) | -0.28(-0.45-  -0.10) |
| Poland | 242(215-266) | 264(234-290) | 5198(4626-5731) | 0.14(-0.06-  0.34) | 0.10(-0.08-  0.28) | -0.12(-0.32-  0.08) |
| Portugal | 102(86.9-116) | 105(89.2-119) | 1988(1707-2256) | -0.11(-0.21-  -0.01) | -0.36(-0.43-  -0.28) | -0.50(-0.59-  -0.42) |
| Puerto Rico | 61.1(47.5-75.1) | 55.1(42.5-67.6) | 1003(783-1236) | -1.87(-2.11-  -1.63) | -2.36(-2.58-  -2.13) | -2.54(-2.74-  -2.33) |
| Qatar | 99.9(75.3-135) | 108(81.3-149) | 2071(1530-2881) | -1.88(-2.58-  -1.18) | -2.01(-2.72-  -1.31) | -2.05(-2.76-  -1.32) |
| Republic of Korea | 221(172-275) | 166(129-203) | 2796(2208-3406) | 0.38(-0.07-  0.83) | -0.84(-1.26-  -0.43) | -1.38(-1.79-  -0.96) |
| Republic of Moldova | 93.6(80.3-109) | 94.2(80.8-109) | 1957(1666-2276) | -0.73(-1.15-  -0.31) | -0.90(-1.31-  -0.49) | -0.96(-1.36-  -0.56) |
| Romania | 149(124-178) | 151(128-177) | 3063(2580-3605) | 1.32(1.09-1.53) | 1.14(0.93-1.35) | 0.95(0.72-1.18) |
| Russian Federation | 136(122-150) | 126(113-139) | 2550(2275-2819) | -0.74(-0.89-  0.60) | -1.09(-1.24-  -0.95) | -1.34(1.52-  -1.17) |
| Rwanda | 50.6(34.6-69.1) | 56.1(38.5-76.5) | 1143(783-1572) | -1.05(-1.35-  -0.76) | -1.02(-1.30-  -0.73) | -1.23(-1.52-  -0.93) |
| Saint Kitts and Nevis | 65.4(53.4-77.8) | 68.5(56.5-80.3) | 1306(1056-1558) | 0.50(0.27-0.72) | 0.34(0.12-0.56) | 0.19(-0.02-0.41) |
| Saint Lucia | 64.5(51.1-78.6) | 68.3(55.1-82.8) | 1237(983-1515) | -1.07(-1.28-  -0.86) | -1.19(-1.42-  -0.96) | -1.39(-1.60-  -1.18) |
| Saint Vincent and the Grenadines | 51.7(44.6-59.8) | 55.6(48.3-64.2) | 1018(876-1186) | -0.62(-0.76-  -0.48) | -0.65(-0.79-  -0.51) | -0.95(-1.09-  -0.82) |
| Samoa | 55.5(41.1-71.6) | 61.1(45.4-78.6) | 1228(905-1589) | -0.22(-0.28-  -0.17) | -0.23(-0.29-  -0.18) | -0.34(-0.41-  -0.28) |
| San Marino | 112(61.4-172) | 101(54.6-155) | 1830(951-2865) | -1.45(-1.86-  -1.03) | -1.64(-2.05-  -1.22) | -1.86(-2.23-  -1.48) |
| Sao Tome and Principe | 125(95.1-166) | 140(106-183) | 2678(2012-3530) | 1.31(1.23-1.39) | 1.32(1.24-1.41) | 1.16(1.10-1.24) |
| Saudi Arabia | 44.3(35.2-55.6) | 48.6(38.9-60.6) | 968(766-1221) | 0.07(-0.15-  0.29) | 0.01(-0.22-  0.23) | -0.04(-0.27-0.19) |
| Senegal | 58.1(43.4-75.8) | 64.9(48.6-84.8) | 1287(956-1698) | 0.73(0.51-0.95) | 0.75(0.53-0.98) | 0.63(0.40-0.85) |
| Serbia | 220(163-287) | 219(163-286) | 4426(3243-5816) | 0.27(0.09-0.45) | -0.02(-0.19-  0.16) | -0.11(-0.30-0.09) |
| Seychelles | 90.9(73.9-111) | 101(81.7-123) | 1876(1501-2312) | -0.92(-1.24-  -0.59) | -0.96(-1.26-  -0.65) | -1.15(-1.41-  -0.88) |
| Sierra Leone | 44.5(31.3-61.2) | 49.6(35.1-68.3) | 998(699-1375) | 0.60(0.35-0.85) | 0.59(0.35-0.83) | 0.61(0.34-0.87) |
| Singapore | 151(128-173) | 115(99.4-131) | 1982(1725-2263) | -1.66(-1.85-  -1.49) | -2.60(-2.76-  -2.43) | -3.02(-3.19-  -2.84) |
| Slovakia | 168(126-215) | 149(112-188) | 2959(2193-3748) | -1.11(-1.18-  -1.04) | -1.40(-1.49-  -1.31) | -1.60(-1.69-  -1.51) |
| Slovenia | 185(150-218) | 172(142-202) | 3305(2710-3886) | -0.15(-0.33-  0.03) | -0.61(-0.79-  -0.43) | -0.86(-1.08-  -0.63) |
| Solomon Islands | 118(90.5-156) | 132(101-172) | 2693(2038-3554) | -0.21(-0.40-  -0.03) | -0.21(-0.39-  -0.02) | -0.31(-0.49-  -0.11) |
| Somalia | 33.4(21.6-58.6) | 36.6(23.9-63.3) | 793(522-1374) | -0.18(-0.25-  -0.12) | -0.19(-0.26-  -0.13) | -0.16(-0.22-  -0.10) |
| South Africa | 115(103-128) | 126(114-141) | 2485(2236-2783) | 0.43(0.10-0.77) | 0.39(0.06-0.73) | 0.33(-0.03-0.68) |
| South Sudan | 51.6(36.9-70.5) | 57.2(41.1-77.2) | 1176(839-1595) | -0.09(-0.16-  -0.03) | -0.05(-0.10-  0.01) | -0.20(-0.29-  -0.11) |
| Spain | 167(141-191) | 141(120-158) | 2676(2315-2999) | -0.24(-0.35-  -0.15) | -0.64(-0.72-  -0.55) | -0.83(-0.90-  -0.75) |
| Sri Lanka | 45.4(28.6-64.3) | 48.1(30.5-67.4) | 950(585-1358) | -0.47(-0.71-  -0.23) | -0.64(-0.88-  -0.39) | -0.69(-0.94-  -0.45) |
| Sudan | 57.4(39.9-82.6) | 64.8(44.7-92.9) | 1267(873-1806) | 0.77(0.68-0.85) | 0.79(0.71-0.87) | 0.64(0.54-0.73) |
| Suriname | 76.7(55.7-104) | 82.9(60.1-112) | 1596(1148-2159) | -0.11(-0.40-  0.18) | -0.16(-0.43-  0.12) | -0.32(-0.61-  -0.02) |
| Sweden | 117(95.8-136) | 124(95.5-136) | 2099(1746-2446) | 0.27(0.06-0.46) | 0.12(-0.04-  0.28) | -0.15(-0.33-0.03) |
| Switzerland | 168(139-191) | 125(105-138) | 2296(1985-2561) | -0.28(-0.41-  -0.14) | -0.68(-0.82-  -0.55) | -0.92(-1.06-  -0.78) |
| Syrian Arab Republic | 70.1(48.9-95.4) | 78.4(55.1-106) | 1490(1018-2067) | -0.45(-0.63-  -0.26) | -0.47(-0.65-  -0.28) | -0.64(-0.83-  -0.45) |
| Taiwan (Province of China) | 164(142-184) | 166(143-184) | 2846(2472-3171) | -0.36(-0.88-  0.17) | -0.49(-0.91-  -0.08) | -1.11(-1.51-  -0.72) |
| Tajikistan | 49.6(34.9-69.8) | 54.3(38.1-75.9) | 1121(795-1557) | -1.60(-1.97-  -1.24) | -1.58(-1.95-  -1.22) | -1.73(-2.09-  -1.37) |
| Thailand | 140(102-184) | 153(111-198) | 2708(1932-3603) | -1.79(-2.02-  -1.56) | -1.88(-2.10-  -1.65) | -2.24(-2.49-  -1.99) |
| Timor-Leste | 95.9(71.1-126) | 107(79.4-139) | 2145(1580-2832) | 0.31(0.16-0.47) | 0.28(0.13-0.44) | 0.22(0.06-0.38) |
| Togo | 53.9(36.4-75.8) | 59.6(39.9-84.2) | 1225(813-1734) | 0.47(0.41-0.53) | 0.45(0.38-0.51) | 0.46(0.39-0.53) |
| Tokelau | 149(110-195) | 165(124-216) | 3131(2299-4124) | 0.11(0.05-0.16) | 0.11(0.05-0.16) | -0.10(-0.16-  -0.04) |
| Tonga | 197(148-253) | 219(166-280) | 4185(3150-5372) | -0.03(-0.26-  0.19) | -0.04(-0.26-  0.18) | -0.15(-0.38-  0.07) |
| Trinidad and Tobago | 54.6(40.5-70.8) | 56.2(42.0-72.5) | 1074(788-1406) | -0.90(-1.01-  -0.79) | -1.09(-1.19-  -0.98) | -1.18(-1.29-  -1.08) |
| Tunisia | 105(68.3-152) | 116(75.1-165) | 2299(1455-3325) | -0.98(-1.16-  -0.81) | -1.02(-1.19-  -0.84) | -1.10(-1.28-  -0.92) |
| Turkey | 201(153-256) | 221(170-281) | 4322(3301-5576) | -1.20(-1.44-  -0.97) | -1.23(-1.47-  -0.98) | -1.45(-1.66-  -1.23) |
| Turkmenistan | 44.1(33.9-56.4) | 47.6(37.3-61.6) | 989(773-1288) | -2.11(-2.49-  -1.72) | -2.10(-2.48-  -1.71) | -2.29(-2.67-  -1.92) |
| Tuvalu | 152(117-199) | 168(130-222) | 3338(2556-4418) | 0.18(0.11-0.25) | 0.21(0.13-0.28) | 0.01(-0.04-  0.05) |
| Uganda | 46.1(33.8-62.2) | 50.9(37.5-68.1) | 1036(755-1398) | -0.04(-0.28-  0.21) | -0.03(-0.27-  0.21) | -0.14(-0.40-  0.12) |
| Ukraine | 92.3(62.6-128) | 82.0(56.2-114) | 1668(1102-2366) | -2.43(-2.57-  -2.29) | -2.62(-2.78-  -2.46) | -2.73(-2.91-  -2.55) |
| United Arab Emirates | 101(74.2-143) | 111(82.4-159) | 2145(1574-3106) | 0.74(0.09-1.39) | 0.76(0.10-1.42) | 0.42(-0.21-1.05) |
| United Kingdom | 234(209-247) | 191(171-201) | 3393(3102-3554) | -0.89(-1.02-  -0.77) | -1.41(-1.50-  -1.31) | -1.78(-1.87-  -1.69) |
| United Republic of Tanzania | 46.1(32.6-62.6) | 51.1(36.7-69.3) | 1035(739-1418) | 0.04(-0.01-  0.09) | 0.06(0.01-0.11) | -0.06(-0.11-  -0.01) |
| United States of America | 236(211-252) | 188(167-200) | 3428(3096-3622) | -1.60(-1.94-  -1.25) | -1.81(-2.11-  -1.50) | -2.19(-2.47-  -1.92) |
| United States Virgin Islands | 59.5(39.2-87.4) | 61.1(40.3-89.8) | 1096(711-1639) | -2.11(-2.42-  -1.80) | -2.23(-2.54-  -1.91) | -2.45(-2.74-  -2.16) |
| Uruguay | 193(174-213) | 201(181-222) | 3936(3569-4330) | -0.39(-0.51-  -0.27) | -0.49(-0.61-  -0.38) | -0.60(-0.73-  -0.47) |
| Uzbekistan | 39.8(32.9-47.9) | 43.2(35.8-51.3) | 887(733-1056) | -2.36(-2.66-  -2.05) | -2.33(-2.64-  -2.03) | -2.56(-2.86-  -2.26) |
| Vanuatu | 121(82.1-191) | 134(92.1-209) | 2684(1831-4199) | -0.02(-0.07-  0.03) | -0.01(-0.06-  0.04) | -0.09(-0.14-  -0.04) |
| Venezuela (Bolivarian Republic of) | 105(79.9-136) | 115(86.8-148) | 2188(1631-2856) | -0.23(-0.40-  -0.05) | -0.26(-0.43-  -0.09) | -0.49(-0.65-  -0.32) |
| Viet Nam | 126(93.6-159) | 136(102-169) | 2678(1976-3399) | -0.11(-0.27-  0.04) | -0.18(-0.34-  -0.02) | -0.43(-0.60-  -0.27) |
| Yemen | 64.2(40.9-101) | 72.1(46.4-113) | 1439(925-2243) | 0.65(0.54-0.76) | 0.69(0.58-0.81) | 0.48(0.37-0.58) |
| Zambia | 68.5(50.8-102) | 75.5(56.1-112) | 1568(1159-2366) | 0.65(0.55-0.75) | 0.66(0.56-0.75) | 0.59(0.48-0.71) |
| Zimbabwe | 88.1(68.9-113) | 97.5(76.5-125) | 2008(1561-2583) | 0.32(0.04-0.60) | 0.34(0.05-0.62) | 0.34(0.06-0.62) |
